# Unconventional receptor functions and location-biased signaling of the lactate GPCR in the nucleus

**DOI:** 10.26508/lsa.202503226

**Published:** 2025-02-04

**Authors:** Mohammad Ali Mohammad Nezhady, Gael Cagnone, Emmanuel Bajon, Prabhas Chaudhari, Monir Modaresinejad, Pierre Hardy, Damien Maggiorani, Christiane Quiniou, Jean-Sébastien Joyal, Christian Beauséjour, Sylvain Chemtob

**Affiliations:** 1 Program in Molecular Biology, Faculty of Medicine, Université de Montréal , Montreal, Canada; 2 Research Center of Centre Hospitalier Universitaire Sainte-Justine , Montreal, Canada; 3 Department of Experimental Medicine, McGill University, Montréal, Canada; 4 Program in Biomedical Sciences, Faculty of Medicine, Université de Montréal , Montreal, Canada; 5 Department of Pharmacology, Université de Montréal , Montreal, Canada

## Abstract

G-protein-coupled receptors (GPCRs) are virtually involved in every physiological process. However, mechanisms for their ability to regulate a vast array of different processes remain elusive. An unconventional functional modality could at least in part account for such diverse involvements but has yet to be explored. We found HCAR1, a multifunctional lactate GPCR, to localize at the nucleus and therein capable of initiating location-biased signaling notably nuclear-ERK and AKT. We discovered that nuclear HCAR1 (N-HCAR1) is directly involved in regulating diverse processes. Specifically, N-HCAR1 binds to protein complexes that are involved in promoting protein translation, ribosomal biogenesis, and DNA-damage repair. N-HCAR1 also interacts with chromatin remodelers to directly regulate gene expression. We show that N-HCAR1 displays a broader transcriptomic signature than its plasma membrane counterpart. Interestingly, exclusion of HCAR1 from the nucleus has the same effect as its complete cellular depletion on tumor growth and metastasis in vivo. These results reveal noncanonical functions for a cell nucleus-localized GPCR that are distinct from traditional receptor modalities and through which HCAR1 can participate in regulating various cellular processes.

## Abstract (precis)

HCAR1 as GPCR for Lactate is highly expressed in many cancers due to the Warburg effect. We show HCAR1 has a nuclear localization where it promotes cancer malignancy through chromatin regulation, DNA damage repair, translational regulation, and intranuclear signaling.

## Introduction

GPCRs are considered the forefront of cellular communication, yet are largely reduced to the role of ligand signal conveyors from the plasma membrane. Meanwhile, there is a surge in discovery of functional intracellular GPCRs. Every membranous organelle has been shown to harbor active GPCRs, either as a primary site of localization or as a result of plasma membrane translocation upon ligand binding (Jong et al, 2018; Mohammad Nezhady et al, 2020). The differential signaling activity of a GPCR from these intracellular organelles as opposed to their signaling output from plasma membrane is generally referred to as location-biased signaling (Mohammad Nezhady et al, 2020). In this context, spatiotemporal coordination of GPCR signaling is determinant (Kwon et al, 2022) and can lead to different outputs even though downstream effectors remain constant (Tsvetanova & von Zastrow, 2014). For example, antagonism of NK_1_R in endosomes is more effective with longer effect in pain relief than targeting this receptor at the plasma membrane (Jensen et al, 2017). Despite showing clinical and translational relevance (Jensen et al, 2017), location-biased signaling remains an understudied concept. Besides location-biased signaling, it has recently been proposed that GPCRs might possess nonsignaling activities. Indeed, a population of PAR2 receptor localizes to nucleus, was shown to interact with SP1 transcription factor, and regulates gene expression (Joyal et al, 2014). Thus, it appears that the location bias of GPCRs permits a diversification of the regulatory roles of GPCRs, either through downstream signaling or through other interactions. However, the latter concept remains unexplored.

Hydroxycarboxylic acid receptor 1 (HCAR1), a GPCR also known as GPR81, is the receptor for lactate (Liu et al, 2009), which is a glycolysis metabolite present at high concentrations in most tumors as a result of the Warburg effect (Vander Heiden et al, 2009). Accordingly, a major focus for this receptor has been placed on cancer studies (Brown et al, 2020; Brown & Ganapathy, 2020; Zhao et al, 2020). Although the Warburg effect associated with marked elevations in lactate concentrations (up to ∼50 mM within the tumor microenvironment) (Liberti & Locasale, 2016; Sun et al, 2017) has been linked to different processes in promoting cancer progression, this feature remains enigmatic due to the paradoxical metabolic switch (Devic, 2016). Remarkably *HCAR1* is overexpressed in numerous cancer cell lines and resected tumors from patients (Roland et al, 2014; Stäubert et al 2015; Lee et al, 2016), and promotes tumor proliferation, metastasis, angiogenesis, survival and immune evasion in vivo (Roland et al, 2014; Lee et al, 2016; Feng et al, 2017). Lactate through HCAR1 promotes DNA-damage repair (Wagner et al, 2015; Wagner et al, 2017) in cancer cells and abolishes IFN-α production in immune cells (Raychaudhuri et al, 2019). Interestingly, although HCAR1 is considered to date a cell surface receptor, its actions were limited when intracellular lactate uptake was inhibited (Wagner et al, 2015; Wagner et al, 2017; Raychaudhuri et al, 2019), suggesting that cell surface HCAR1 signaling was not determinant for its functions. Thus, mechanisms to explain such multidimensional involvement of HCAR1 in cancer biology are lacking; in this context, an intracellular mode of action along with possible non-traditional signaling activities of the receptor should be accounted for.

In the present study, we show that HCAR1 has a nuclear localization and decipher its topology on the nuclear membranes. We show that nuclear HCAR1 (N-HCAR1) is capable of initiating G_α_ and G_βγ_- mediated intranuclear signaling, and using bottom-up high-throughput omics studies demonstrate that N-HCAR1 promotes various processes through different nontraditional receptor mechanisms involving formation of protein complexes inside the nucleus that promote protein translation and DNA-damage repair. N-HCAR1 is found to regulate a broader transcriptomic signature than its plasma membrane counterpart, emphasizing that N-HCAR1 functional output is larger than its plasma membrane localized counterpart. Cellular effects of N-HCAR1 which translate into cell proliferation, survival and migration in vivo unveil importance of N-HCAR1 in promoting a variety of roles in cancer malignancy.

## Results

### HCAR1 displays a nuclear localization pattern dependent upon the third intracellular loop domain and S305 phosphorylation site

**Figure 1.**
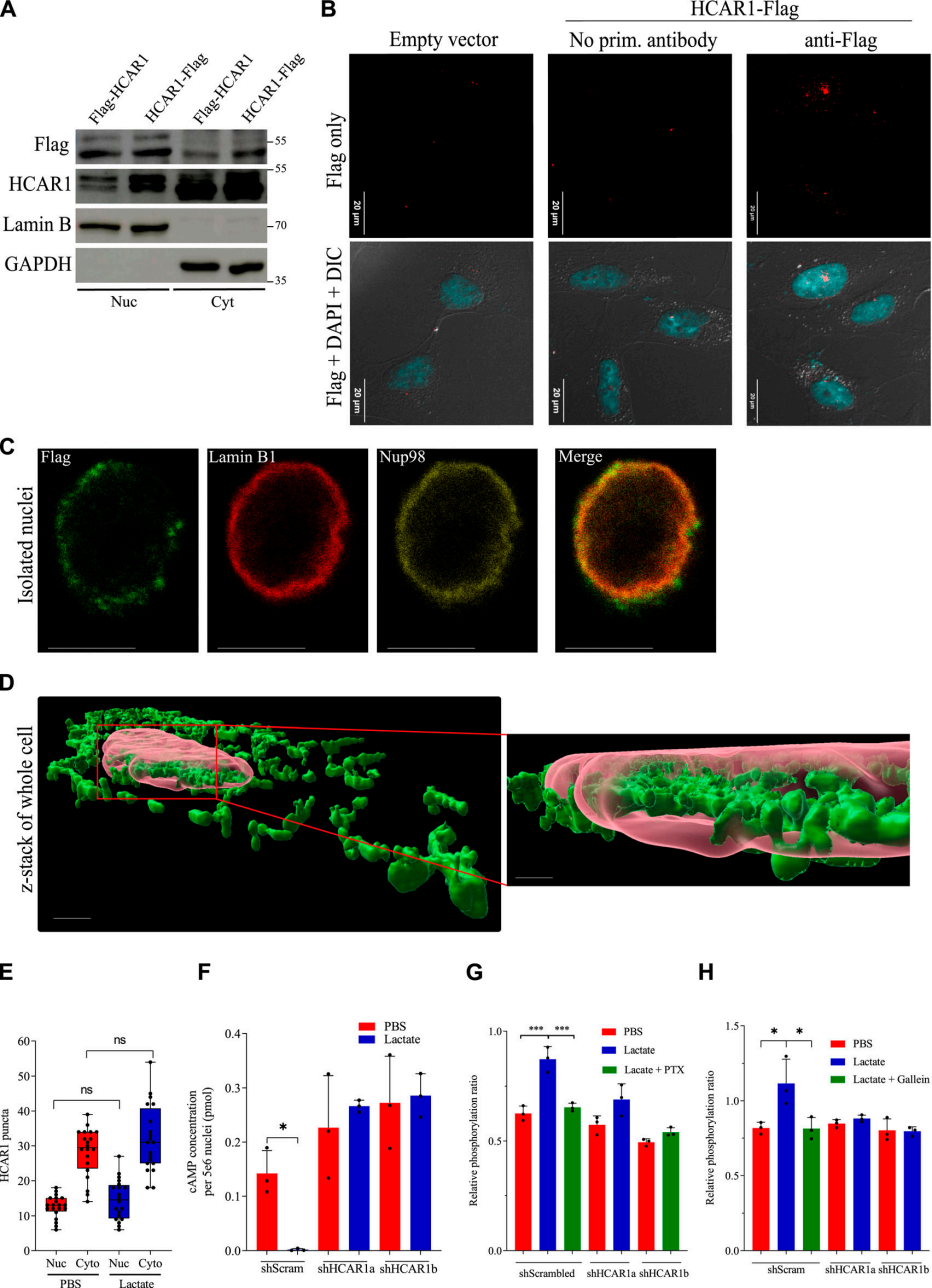
HCAR1 is present in the nucleus and induces nuclear location-biased signaling. **(A)** Western blot analysis of biochemical fractionation from cells transfected with C & N-terminally flag-tagged HCAR1. Lamin B and GAPDH were used to confirm pure isolation of nuclei. **(B)** Wide-field immunofluorescence microscopy of C-terminal flagged-tagged HCAR1. The image is 4-planes of z-stacks with a step of 600 nm. **(C)** Confocal microscopy of C-terminally flag-tagged HCAR1 in isolated nuclei. **(D)** 3D rendering of z-stacked confocal images of C-terminally flag-tagged HCAR1 whole cells. Transparent red is Lamin B and green is anti-flag. Z-stack are 200 nm layers. (scale bar in the right image is 1.5 μm). **(E)** Quantification of HCAR1 from TEM images of PBS- and lactate-treated cells (10 mM for 1 h; Fig S2A–F) from 20 cells in two biological replicate experiments. (Nuc = nuclear; Cyto = Cytoplasmic fractions). **(F)** cAMP level in isolated nuclei from scrambled shRNA (endogenously expressing HCAR1) or two different HCAR1 KD cells with PBS or lactate treatment (10 mM for 10 min). The cAMP concentration is presented in picomole per 5 million nuclei. **(G, H)** ELISA analysis of ERK (G) and AKT (H) phosphorylation rates in isolated nuclei from scrambled shRNA (endogenously expressing HCAR1) or two different HCAR1 KD cells with PBS or lactate treatment (10 mM for 15 min). PTX or Gallein treatment of cells was performed prior to nuclei isolation. Scale bars are 5 μm, unless otherwise indicated. Source data are available for this figure.

**Figure S1.**
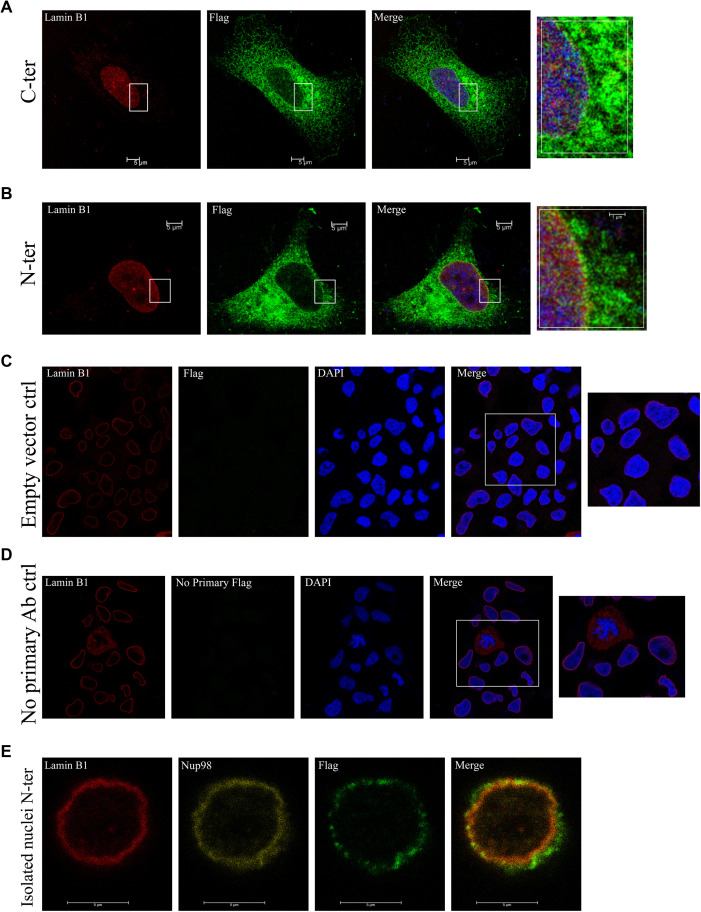
Extra validations of HCAR1 nuclear localization and controls. **(A, B)** Immunofluorescence confocal microscopy of C and N-terminally flag-tagged HCAR1 in HeLa cells. **(C, D)** Negative control experiments for HCAR1 localization with empty vector containing flag tag (C), and no primary antibody staining control (D) in immunofluorescence confocal imaging. **(E)** Immunofluorescence confocal imaging of N-terminally flag-tagged HCAR1 in isolated nuclei.

**Figure S2.**
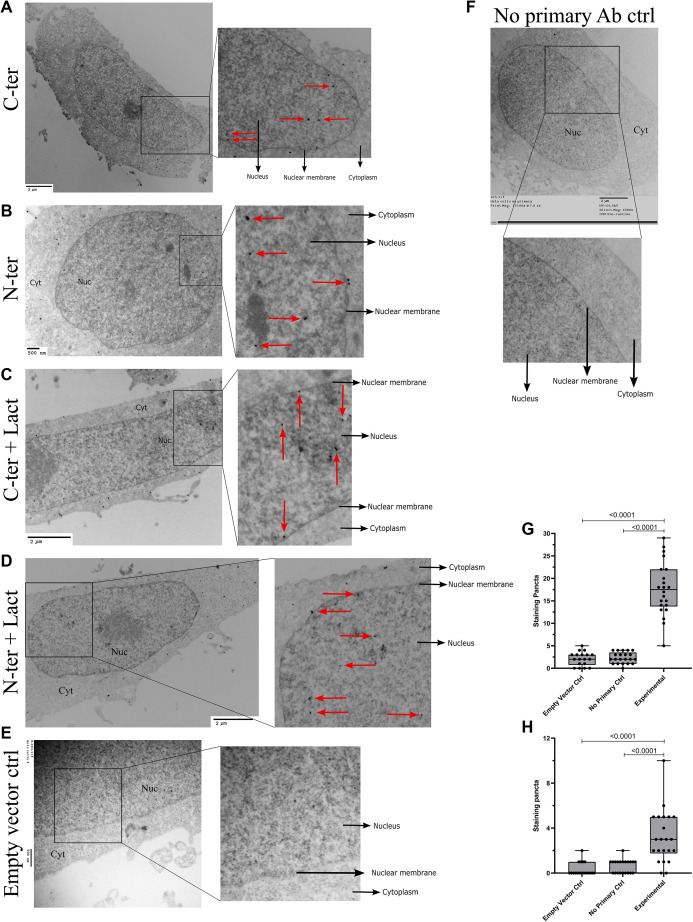
Extra validations of HCAR1 nuclear localization and controls with TEM. **(A, B)** TEM graphs from N- and C-terminally flag-tagged HCAR1. **(C, D)** TEM images of N- and C-terminally flag-tagged HCAR1 with lactate treated (10 mM for 1 h) cells. **(E, F)** Negative control experiments for HCAR1 localization with empty vector containing flag tag (E), and no primary antibody staining control (F) in TEM images. **(G, H)** Quantification of intranuclear (G) and nuclear membrane (H) immunosignals in the negative controls (empty vector and no primary control samples) and experimental samples show the specificity of staining both in the intranuclear and on the nuclear membrane. All images are in HeLa cells.

**Figure S3.**
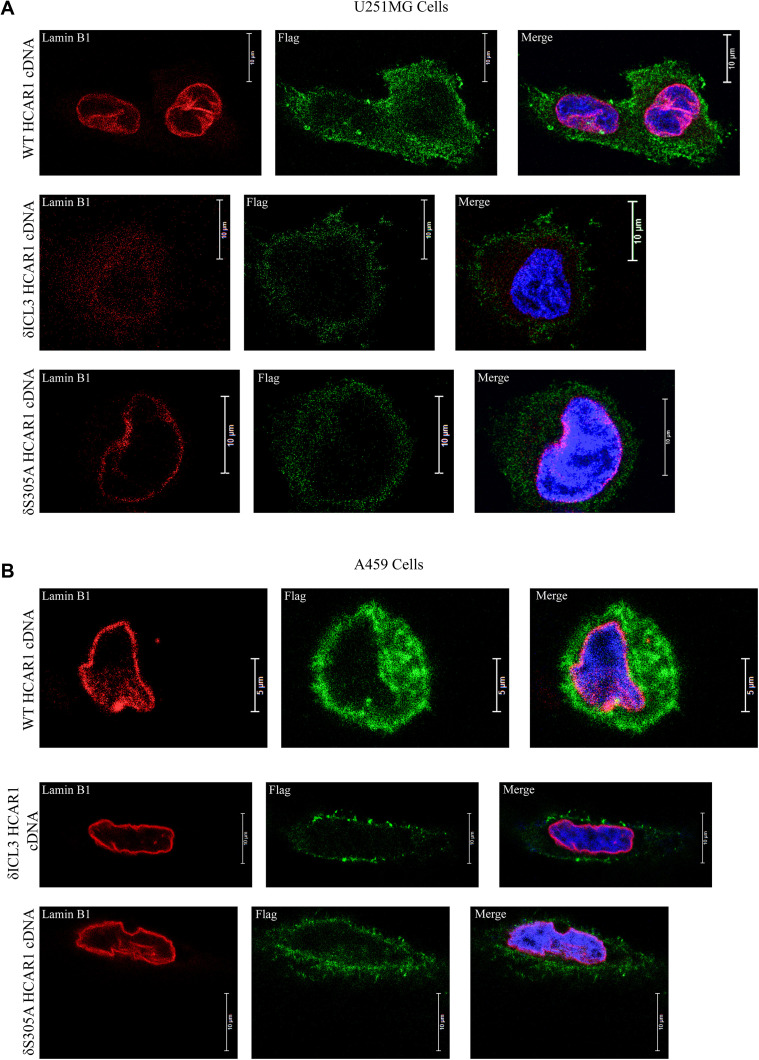
HCAR1 localization in other cell lines. **(A)** Immunofluorescence confocal microscopy of C-terminally flag-tagged HCAR1 in U251MG cells with WT HCAR1, δICL3 HCAR1, and δS305A HCAR1 cells. **(B)** Immunofluorescence confocal microscopy of C-terminally flag-tagged HCAR1 in A549 cells with WT HCAR1, δICL3 HCAR1, and δS305A HCAR1 cells.

We generated stable HeLa cell lines expressing either C-terminal or N-terminal Flag-tagged HCAR1 enabling to use various methods to ascertain its subcellular localization and ensuing functions. Complete nuclear isolation upon biochemical cell fractionation revealed abundant HCAR1 at the nucleus, as well as in the cytoplasm (Fig 1A). Immunofluorescent staining with wide-field microscopy (Fig 1B) showed nuclear localization and confocal microscopy with Lamin B1 as inner nuclear membrane marker exhibited clear HCAR1 colocalization with Lamin B1 in intact cells (Fig S1A–E) and isolated nuclei (Fig 1C). Strikingly, HCAR1 was also detectable inside the nucleus (Figs 1B and C and S1A and B). 3D rendering of z-stacked confocal images clearly showed HCAR1 is present inside the nucleus (Fig 1D). Moreover, electron microscopy using immunogold staining of HCAR1 confirmed nuclear envelope and intranuclear HCAR1 distribution (Fig S2A–H). Quantification of electron microscopy showed nearly one-third of cellular HCAR1 localized at the nucleus in unstimulated cells (Fig 1E). Nuclear localization of HCAR1 was also detected in U251MG and A549 HCAR1-expressing cells (Fig S3A and B).

**Figure S4.**
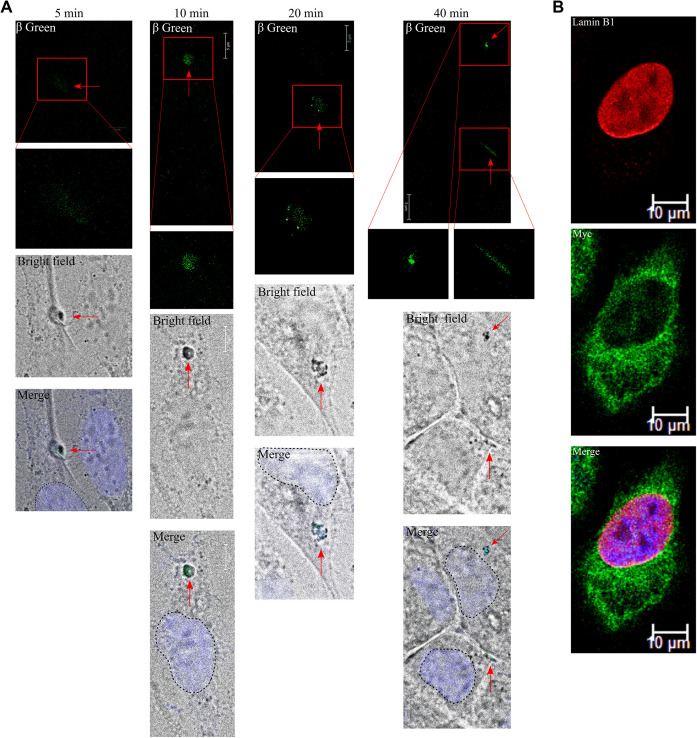
Pulse-chase assay with FAP for HCAR1 shows no translocation from PM. **(A)** Confocal imaging of pulse-chase FAP system with HCAR1 in HeLa cells using impermeant green fluorogen followed by lactate treatment (10 mM) for 5, 10, 20, and 40 min. **(B)** Immunofluorescence confocal imaging pMFAP-β1 FAP construct with C-terminally Myc-tagged HCAR1 in HeLa cells.

Treatment of cells with lactate did not alter the nuclear ratio of HCAR1, indicating that ligand stimulation does not lead to translocation from plasma membrane to the nucleus (Figs 1E and S2C and D); as is the case for some other nuclear GPCRs (Joyal et al, 2014). To ensure this biologic process, we devised a pulse-chase experiment using fluorogen-activating peptide (FAP) technology utilizing cell impermeable fluorogen (Fisher et al, 2014). Activation of the receptor with lactate, triggered HCAR1 internalization within 5 min; HCAR1-containing endosomes were tracked in the cytoplasm for up to 40 min, after which they were no longer detected (either because of recycling to the plasma membrane or endosomal degradation) (Fig S4A). Whereas nuclear localization of HCAR1 from plasma membrane in our FAP system was not observed, the chimeric receptor was hitherto present in the nucleus prior to lactate stimulation (Fig S4B). Hence, HCAR1 does not translocate from plasma membrane to the nucleus upon ligand stimulation, and is de facto localized at the nucleus.

Lastly, to ascertain whether the endogenous and functional HCAR1 is present in the nucleus, we isolated intact nuclei (Fig 1A) of WT cells endogenously expressing HCAR1 and stimulated them with lactate and measured nuclear cAMP levels. Lactate treatment (10 mM for 10 min) of nuclei isolated from endogenously HCAR1-expressing HeLa cells significantly decreased cAMP levels. On the other hand, nuclei isolated from cells knocked down (KD) of HCAR1 (using two distinct shRNAs; Source Data 2) did not respond to lactate. This indicates that endogenous and functional HCAR1 is present in the nucleus and is able to induce intranuclear signaling that reduces nuclear cAMP level.

**Figure S5.**
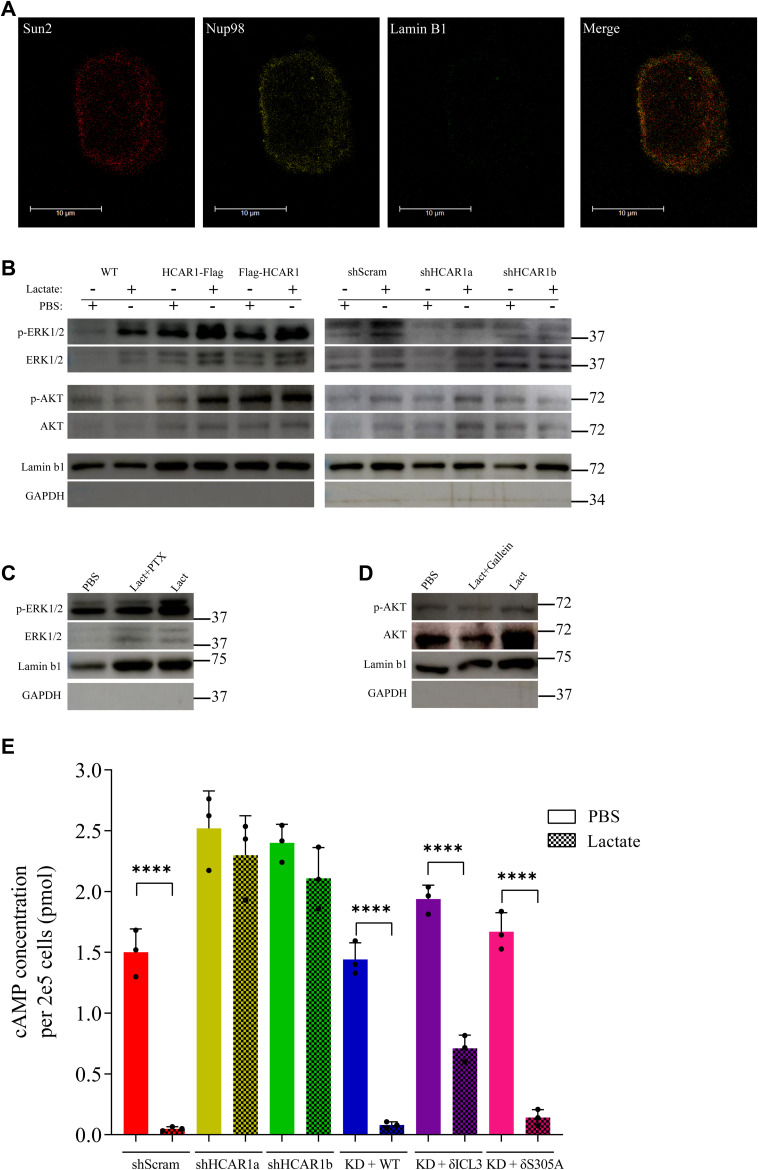
Validations for Fig 2. **(A)** Immunofluorescence confocal imaging of isolated nuclei with selective ONM permeabilization with intact INM. Detection of Sun2 C-terminus indicates INM permeabilization and the absence of Lamin B1 signal indicates intact non-permeabilized INM. **(B)** Western-blot analysis on isolated nuclei from WT cells, cells overexpressing C- and N-terminally tagged HCAR1, shScrambled or two HCAR1 KD HeLa cells. Isolated nuclei were stimulated with PBS or lactate (10 mM for 15 min). **(C, D)** Western-blot analysis on isolated nuclei from shScrambled HeLa cells from different treatments. **(E)** cAMP level in whole cells with PBS or lactate treatment (10 mM for 10 min). The cAMP concentration is presented in picomole per 2 × 10^5^ cells. The decrease in the cAMP level with the mutant rescues show that the signaling activity of the mutant HCAR1 from the plasma membrane is largely intact.

Lactate also induced ERK1/2 and AKT phosphorylation in isolated nuclei from cells expressing endogenous HCAR1, but not in nuclei of cells KD of HCAR1 (Figs 1G and H and S5B). ERK1/2 and AKT phosphorylation were respectively inhibited by pertussis toxin and gallein which accordingly inhibit G_αi_ and G_βγ_ (Figs 1G and H and S5C and D); consistent with report of downstream effectors of GPCR signaling at the nucleus (Jong et al, 2018; Mohammad Nezhady et al, 2020). Together, these findings confirm functional G-protein-coupling (G_αi_ & G_βγ_) of endogenous HCAR1 receptor at the nucleus, elucidating nuclear location-biased signaling of endogenous HCAR1.

### Intranuclear signaling of N-HCAR1 promotes cellular proliferation and survival

**Figure 2.**
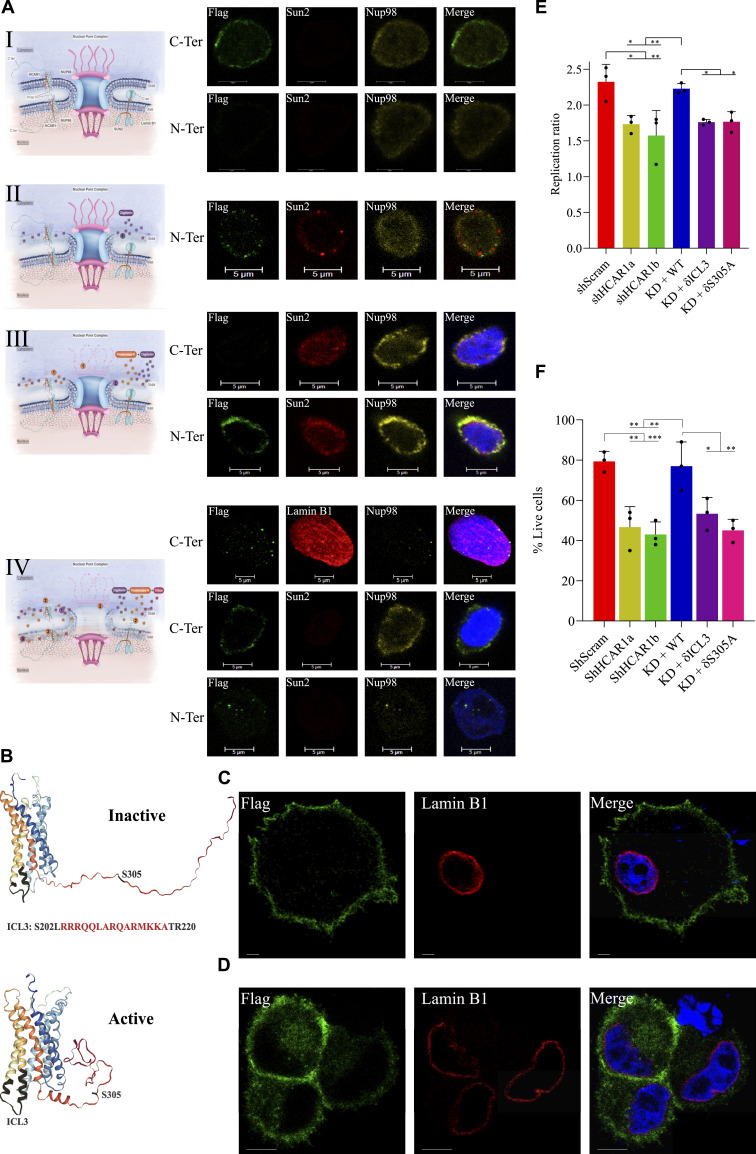
Intranuclear signaling of N-HCAR1 promotes cellular proliferation and survival. **(A)** Confocal images of nuclei isolated from cells expressing C-ter or N-ter flag-tagged HCAR1. (I) intact nuclei, (II) ONM permeabilized nuclei with intact INM, (III) nuclei with surface protein digestion and then ONM permeabilization with intact INM, (IV) ONM permeabilized nuclei with intact INM was treated with PK to digest proteins on the ONM and nuclear lumen, and after washing PK, nuclei were treated with triton to permeabilize INM. Notice loss of Sun2 indicating digestion of luminal proteins. **(B)** 3D modeling of HCAR1 in inactive and active conformations by GPCRM. The black highlights indicate the spanning regions for ICL3 (deletion of RRRQQLARQARMKKA) domain and S305. **(C, D)** Confocal microscopy of C-terminally flag-tagged HCAR1 with ICL3 deletion (C) and S305A substitution mutation (D). **(E)** Cell proliferation rate in scrambled shRNA, two different HCAR1 KD cells, WT-rescue and nuclear HCAR1 KD cell lines. **(F)** Cellular survival rate in 5FU-treated cells. Data are mean ± s.d. from n ≥ 3 biological replicates. Analysis of variance (ANOVA) was followed by Bonferroni post hoc correction test with *P < 0.05, **P < 0.01, ***P < 0.0001 significance levels. Scale bars are 5 μm, unless otherwise indicated. Source data are available for this figure.

Understanding the topology of HCAR1 in nuclear membranes enables better comprehending the cellular location of signaling domains. We observed the intranuclear signaling of HCAR1, but the orientation of nuclear GPCRs for such intranuclear signaling has never been shown. We determined the orientation of HCAR1 on both outer and inner nuclear membranes (ONM, INM, respectively) of the nuclear envelope. Immunofluorescence staining (of Flag) of intact (non-permeabilized) nuclei (Joyal et al, 2014) isolated from HCAR1 C-terminus Flag-tagged expressing cells indicates that the C-terminus of the receptor is oriented towards the cytoplasm on the ONM (Fig 2A-I); whereas N-terminus Flag-tagged HCAR1 in intact nuclei did not reveal staining, consistent with the suggestion that the N-terminus of the receptor resides within the nuclear envelope (Fig 2A-I). This orientation was ascertained by devising a protocol which selectively permeabilizes the ONM, whereas keeping the INM intact. For this purpose we used a combination of three proteins as markers located in different parts of the nuclear envelope: (a) NUP98 - detectable across the nuclear membrane (Xu & Powers, 2009); (b) the C-terminus of SUN2 - a luminal marker (Chang et al, 2015); (c) Lamin B1 - located on the nuclear side of the INM. Selective permeabilization of the ONM allowing antibody access and retaining intact INM (Fig S5A) using (mild detergent) 0.0008% digitonin (Ledet et al, 2021) allowed us to detect the N-terminus Flag-tagged HCAR1, consistent with its luminal nuclear envelope localization (Fig 2A-II). In addition, treatment of the intact nuclei with proteinase K (PK) to remove the cytoplasm-facing C-terminus of HCAR1, followed by permeabilization of the ONM, revealed the absence of the C-terminus in the lumen whereas preserving the signal for the nuclear envelope lumen-localized N-terminus (Fig 2A-III). We then permeabilized the ONM, followed by sequential treatment of nuclei with PK and permeabilization of the INM. Under these conditions, we could again observe HCAR1 C-terminus staining co-localized with Lamin B1 (Fig 2A-IV), whereas the N-terminus in this condition was only detected inside the nucleus. Altogether, these experiments reveal that the C-terminus of HCAR1 at the ONM orients within the cytoplasm, whereas at the INM it has analogous conformation to that at the plasma membrane to initiate signaling cascade into the nucleus (Fig 1F–H), as nuclear envelope membranes are known to contain conventional GPCR signaling machinery (Gobeil et al, 2006).

Next, to determine the mechanism of nuclear localization of N-HCAR1, we analyzed its sequence and 3D model of the receptor in an attempt to determine HCAR1 domains necessary for nuclear localization (Fig 2B) and found that although there is no classical NLS in HCAR1 sequence, there are predicated bipartite NLS in intracellular loop 3 (ICL3), and the C-terminus of the receptor (Kosugi et al, 2009). Corresponding truncation in ICL3 completely abolished nuclear localization and cytoplasmic staining (Figs 2C and S3A and B). A phosphorylation site in the NLS of the C-terminus (Source Data 1) (Hornbeck et al, 2015), prompted us to determine its potential role in localization. Single point substitution of S305 to alanine at the C-terminus led to nuclear exclusion of HCAR1 (but retained cytoplasmic staining; Figs 2D and S3A and B). These findings suggest a scaffolding role of ICL3 and posttranslational phosphorylation of S305 are required for HCAR1 nuclear localization.

**Figure S6.**
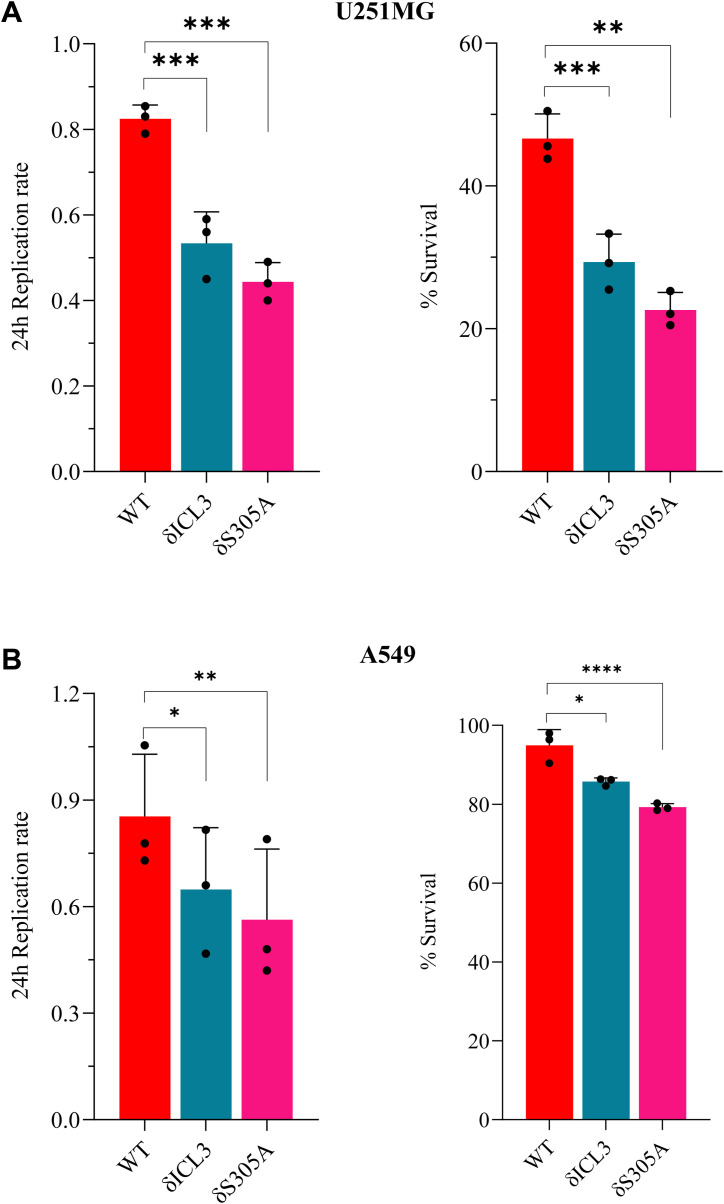
N-HCAR1 promotes proliferation and survival in other cell lines. **(A, B)** Homeostatic proliferation rate (left panel) and survival rate in 5FU-treated cells (right panel) in U251MG (A) and A549 (B) cell lines. Both U251MG and A549 cell lines are expressing lower levels of endogenous *HCAR1* (see Source Data for Figure 2.1 e), so we generated stable cell lines over-expressing either WT HCAR1, or nuclear-excluded δICL3 HCAR1 and δS305A HCAR1 in these two cell lines.

We used these two mutant versions to elucidate the cellular effects of nuclear location-biased signaling of endogenous HCAR1. Since we saw ERK1/2 and AKT signaling (Fig 1G and H) and these pathways modulate proliferation and survival in cancer cells (Martelli et al, 2012; Maik-Rachline et al, 2019), we measured homeostatic cell proliferation rate and cell survival upon 5-Fluorouracil (5-FU) challenge in cells containing or not HCAR1 at the nucleus. Nuclear HCAR1 (N-HCAR1) containing cells include WT cells endogenously expressing HCAR1 with scrambled shRNA (shScram), and HCAR1 KD cells that are rescued with RNAi-resistant WT *HCAR1* (referred to as “WT rescue” cells; Source Data 2A–D). Cells depleted of N-HCAR1 are HCAR1 KD cells rescued with RNAi-resistant *HCAR1* constructs containing δICL3 or δS305A mutations (referred to as N-HCAR1 KD cells; Source Data 2A–D). Whereas all ectopically HCAR1-expressing cells had the same expression level as the endogenous HCAR1 (Source Data 2A–D), HeLa cells harboring WT HCAR1 exhibited higher proliferation and survival rate compared with total HCAR1 KD (un-rescued) and N-HCAR1 KD cells (Fig 2E and F). Interestingly, the magnitude of cell proliferation and survival observed upon exclusion of HCAR1 from the nucleus (as seen with the δICL3 and the S305A rescues) was similar to that of cells totally depleted of HCAR1. Importantly, the mutant versions of the HCAR1 preserved their signaling activity (Fig S5E). Nucleus-excluded HCAR1 mutations caused similar effects in U251MG and A549 cancer cells (Fig S6A and B; Source Data 2E). Hence, nuclear location-biased signaling of HCAR1 promotes proliferation and survival in cancer cells.

### N-HCAR1 interactome discloses unconventional receptor functions in protein translation and DNA damage repair

**Figure S7.**
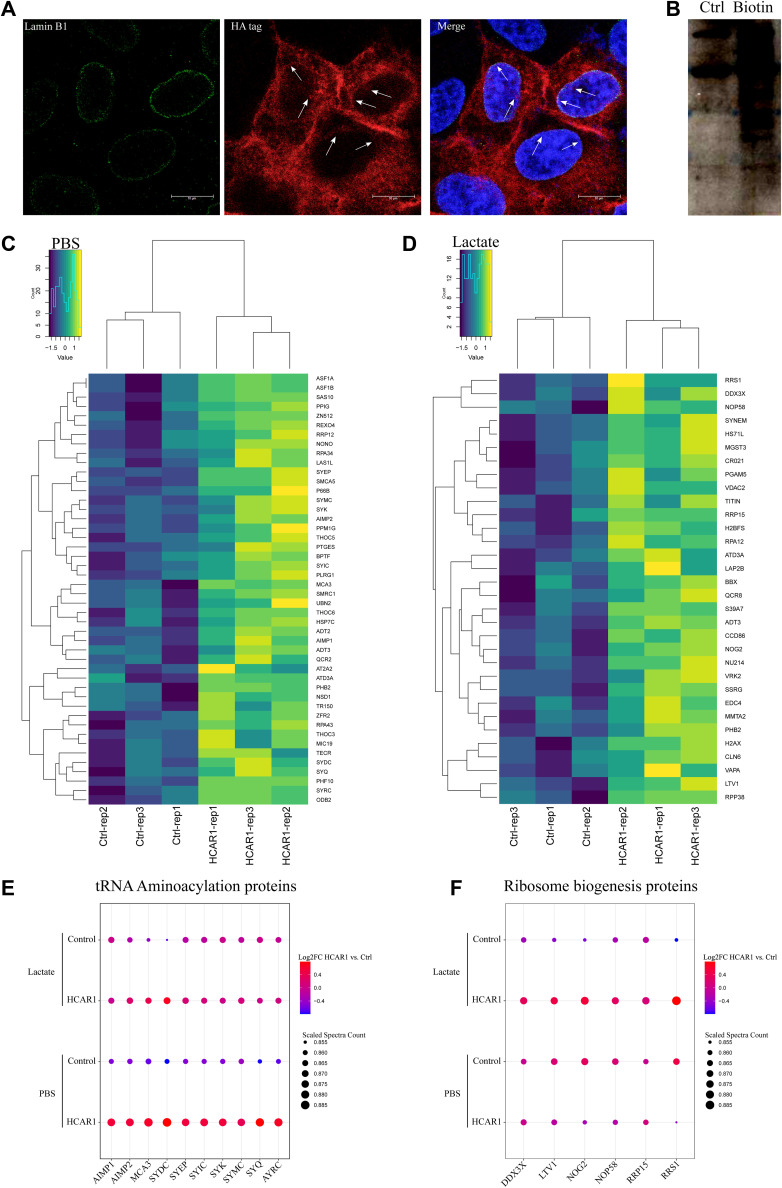
Controls and extra validations of Fig 3. **(A)** Immunofluorescence confocal imaging of HA-tagged HCAR1-BirA construct shows same localization pattern (on the nuclear membrane and in inside the nucleus) for HCAR1-BirA fusion protein as the WT HCAR1 in HeLa cells. **(B)** Western blot analysis with streptavidin-HRP on biotinylated whole cell lysate from PBS or Biotin-treated cells. **(C, D)** Heatmaps showing enrichment of proteins with HCAR1 based on Log_2_ fold change and *P*-value in isolated nuclei of PBS (C) or lactate-treated (D) cells with biotin. Control samples are from stable cell lines expressing empty Bio-ID vector, expressing only BirA. **(E)** Enrichment dot plot graph showing proteins enriched in tRNA aminoacylation pathway in PBS and lactate-treated cells compared with control cells. **(F)** Enrichment dot plot graph showing proteins enriched in ribosome biogenesis pathway in PBS and lactate treated cells compared with control cells.

**Figure 3.**
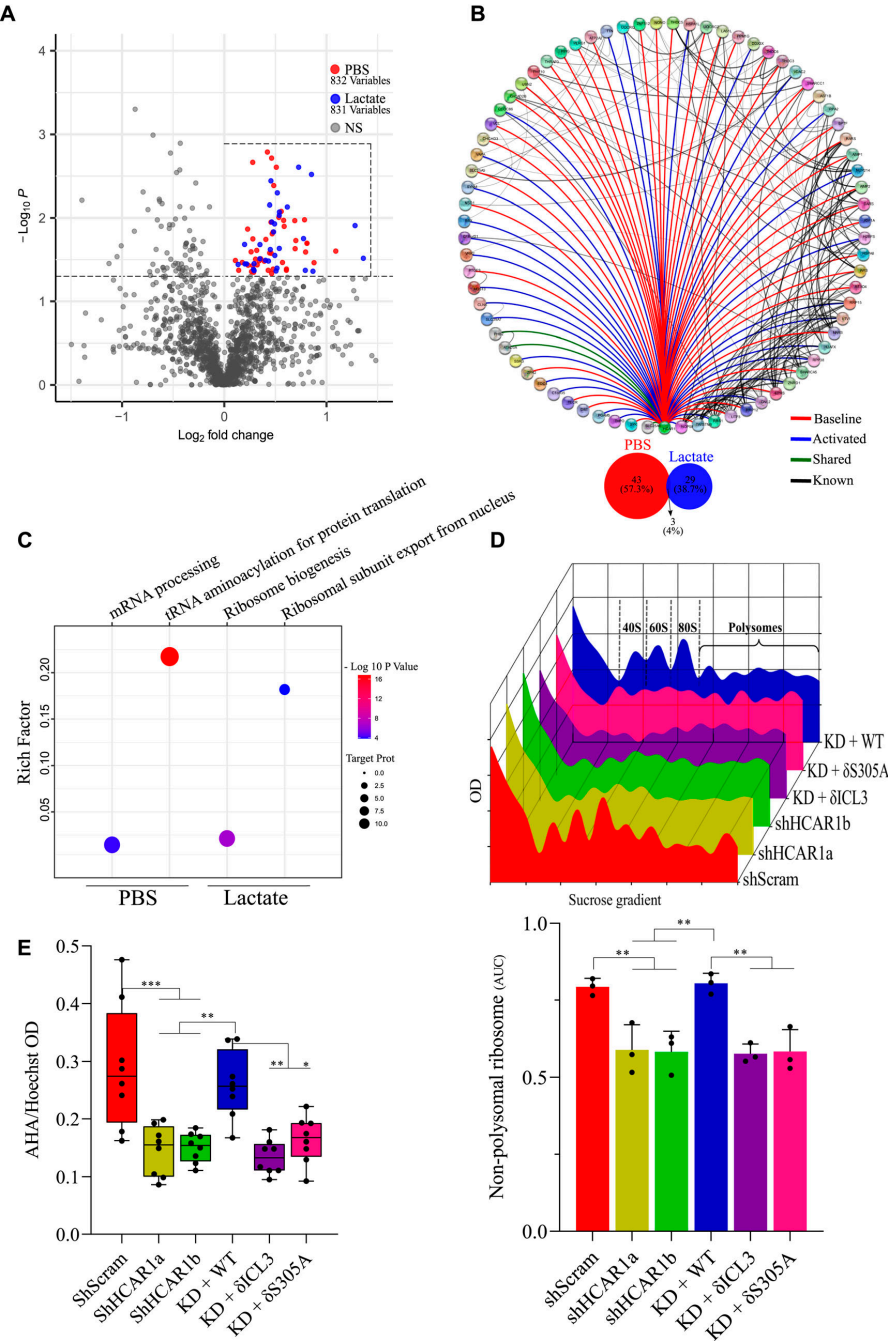
N-HCAR1 interactome is enriched for proteins involved in translation processes and N-HCAR1 promotes protein translation rate. **(A)** Volcano plot representing proteins significantly interacting with N-HCAR1. Plot shows protein abundance (log_2_ fold change) versus significance (−log_10_ *P*-value) in isolated nuclei of HCAR1-BirA expressing cells relative to BirA alone. Significantly enriched proteins in the upper right quadrant (proteins within the dashed square) in both PBS and lactate treated (10 mM for 24 h) samples are selected for subsequent analysis. **(B)** Interactome map of N-HCAR1 in both PBS and lactate treated cells. Red lines indicate interactions of enriched proteins with HCAR1 when treated with PBS, blue lines indicate interactions with HCAR1 when treated with lactate, green lines indicate interactions in both cases, and black lines represents already established interactions based on STRING. The bottom Venn diagram shows unique and overlapping significantly enriched proteins in PBS and lactate treated samples. **(C)** Enrichment dot plot of proteins in panel b based on gene ontology molecular functions (Panther). **(D)** Upper panel: representative sucrose gradient ribosomal profiling for scrambled shRNA, total and nuclear HCAR1 KD, and WT-rescue cells. Lower panel: normalized measurement of the upper panel for area under the curve (AUC) of the monosomes (40S, 60S, and 80S ribosomal subunits). **(E)** Protein translation rate with methionine incorporation rate measurement. Methionine incorporation rate (L-azidohomoalanine; AHA) was adjusted to the number of cells (Hoechst). Data are mean ± s.d. from n ≥ 3 biological replicates. ANOVA was followed by Bonferroni post hoc correction test with *P < 0.05, **P < 0.01, ***P < 0.0001 significance levels. Source data are available for this figure.

**Figure S8.**
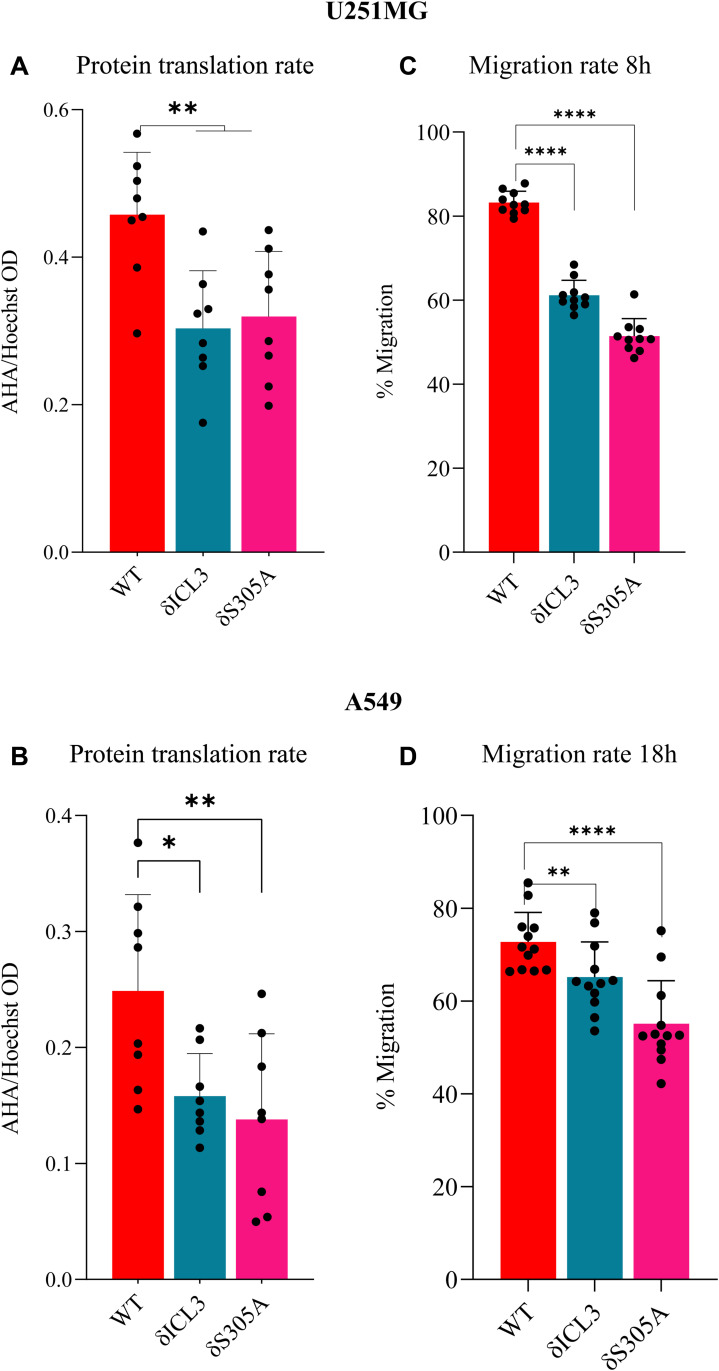
N-HCAR1 promotes protein translation and migration rates in other cell lines. **(A, B)** Protein translation rate with methionine incorporation rate measurement in U251MG (A) and A549 (B) cell lines. Methionine incorporation rate (AHA) was adjusted to the number of cells (Hoechst). **(C, D)** Scratch assay to measure the migration rate in U251MG (C) and A549 (D) cell lines. Migration rate was measured 8 h post-scratch in U251MG cell lines and 18 h post-scratch in A549 cell lines.

Presence of GPCRs inside the nucleus has been reported including by us (Gobeil et al, 2003; Di Benedetto et al, 2014; Joyal et al, 2014; Bhosle et al, 2016), yet their roles independent of membrane-bound G proteins are not known. Hence detection of HCAR1 inside the nucleus prompted us to investigate spatiotemporal interactome of N-HCAR1. We used the Bio-ID system (Kim et al, 2016) to construct HCAR1-Bio-ID fusion protein which again revealed the expected nuclear localization of HA-tagged HCAR1 (Fig S7A and B). Cells were treated or not with lactate followed by nuclear isolation. We purified the biotinylated proteome of isolated nuclei and subjected them to mass spectrometry (Fig 3A). Surprisingly, different proteins found in the interactome of N-HCAR1 are not classical GPCR signaling modulators (Figs 3B and S7C and D; Supplemental Data 1; Table S1), suggesting potential involvement of N-HCAR1 in functions other than canonical receptor-mediated signaling. There was a clear distinction in the interactome of N-HCAR1 stimulated or not with lactate (Figs 3B and S7C and D), suggesting that different conformations of N-HCAR1 participate in distinct protein complexes (Fig 3B). The protein interactome of N-HCAR1 after lactate treatment was enriched for ribosomal regulatory processes (Figs 3C and S7F). Experiments using sucrose gradient ribosomal profiling further revealed that HCAR1 total KD and N-HCAR1 KD cells have a lower content of non-polysomal ribosomes (Fig 3D). The interactome of N-HCAR1 isolated from cells untreated with lactate was particularly enriched for proteins mediating tRNA aminoacylation involved in protein translation (Figs 3C and S7E). Concordantly, quantification of methionine incorporation rate revealed that protein translation was decreased in HCAR1 KD and N-HCAR1 KD HeLa cells compared with cells with intact HCAR1 (Fig 3E). Similar observations on protein translation were made in U251MG and A549 cells (Fig S8A and B).

Supplemental Data 1. N-HCAR1 BioID interactome dataset.

Table S1. List of proteins interacting with N-HCAR1 from BioID experiment.

**Figure 4.**
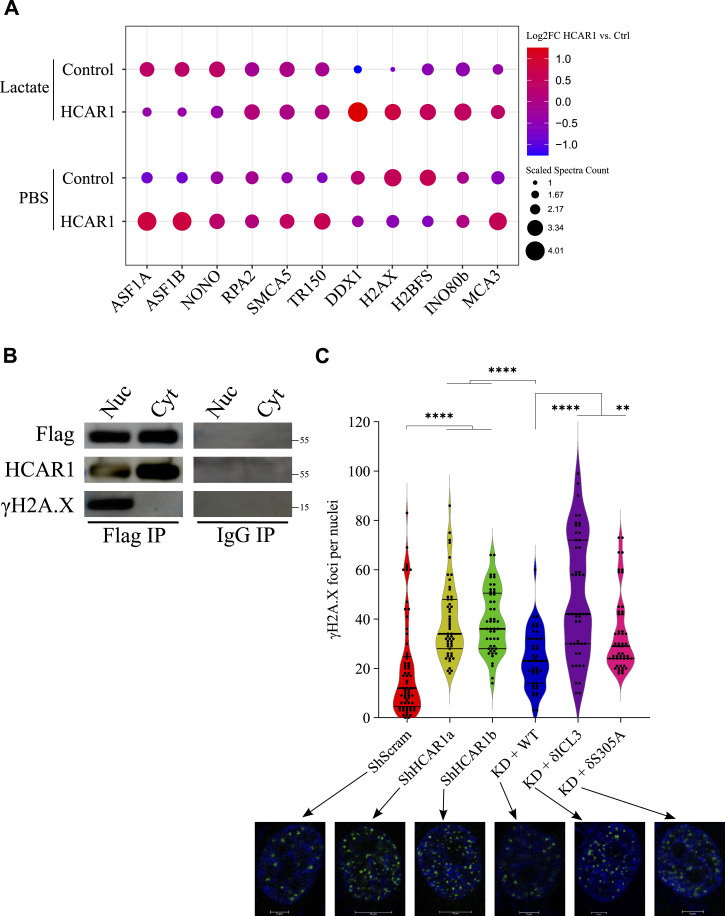
N-HCAR1 with its interactome promotes DNA damage repair. **(A)** Dot plot of enriched proteins interacting with HCAR1 that are involved in DNA damage repair. **(B)** Validation of BioID mass spectrometry for interaction of HCAR1 with H2AX (from Fig 3B). Co-immunoprecipitation of γH2AX with HCAR1 or IgG in fractionated cells. (Nuc = nuclear; Cyt = Cytoplasmic fractions). **(C)** Irradiated cells were let to recover for 4 h and the amount of DNA damage was measured with γH2AX foci. Each dot represents the number of γH2AX foci per nucleus, in four separate experiments. Underneath are the representative nuclei of irradiated cells with confocal imaging of γH2AX staining. Data are mean ± SD from n = 4 biological replicates. ANOVA was followed by Bonferroni post hoc correction test with *P < 0.05, **P < 0.01, ***P < 0.0001 significance levels.

Strikingly, the interactome of N-HCAR1 with and without lactate also revealed components of the DNA damage repair machinery, including the dominant DNA damage marker H2AX (Fig 4A), consistent with the proposed role of HCAR1 in DNA damage response (DDR) (Wagner et al, 2017). We validated the interaction of N-HCAR1 with H2AX from our BioID mass spectrometry data with co-immunoprecipitation (Fig 4B). We thus proceeded to irradiate cultured HeLa cells and measured ensuing γH2AX foci number as a proxy for DDR (Fig 4C). WT and WT HCAR1-rescued cells displayed lower number of γH2AX foci compared to HCAR1 KD and N-HCAR1 KD cells, suggesting nuclei devoid of HCAR1 have limited DNA damage repair capacity (Fig 4C). Thus, the functional effects of N-HCAR1 activity identified from the BioID data were corroborated based on the functional assays in our system. Together these data show that N-HCAR1 interacts with nonclassical GPCR effectors in the nucleus to promote protein translation and DNA damage repair.

### N-HCAR1 is involved in direct gene regulatory function by interacting with chromatin remodelers

**Figure 5.**
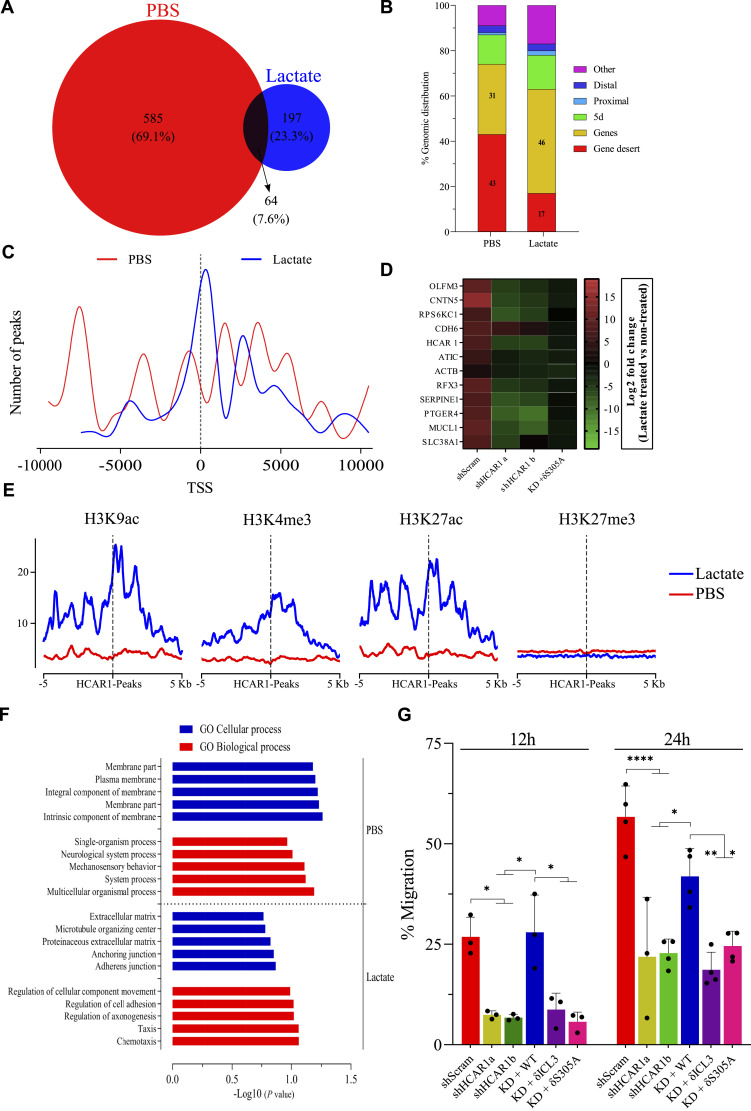
HCAR1 genome-wide interactions show enrichment for genes promoting migration. **(A, B, C)** ChIP-seq of HCAR1 from PBS or lactate-treated (10 mM for 1 h) cells from quadruplicate samples. For controls and validations see Source Data for Figure 5. **(A)** Venn diagram representing the number of genes associated with HCAR1 in each treatment. **(B)** Genomic distribution of HCAR1 in each treatment. Genes (exon or intron), proximal (2 kb upstream of TSS), distal (between 2 and 10 kb upstream of TSS), 5d (between 10 and 100 kb upstream of TSS), Gene desert (≥100 kb up or down stream of TSS), others (anything else). **(C)** Normalized number of HCAR1 peaks around TSS of genes. **(D)** qRT-PCR for the top 4 genes in each section of the Venn diagram (panel a). Expression levels are presented as Log_2_ fold changes of lactate treated (10 mM for 6 h) cells over PBS treatment (n = 4). **(E)** Co-alignment of histone marks from encode project from HeLa cells over HCAR1 peaks. **(F)** Ontological analysis of HCAR1-bound genes in PBS- and lactate-treated samples. **(G)** Scratch assay to measure the migration rate of cells (n = 3). Data in panel (D, G) are mean ± SD from biological replicates. Their ANOVA was followed by Bonferroni post hoc correction test with *P < 0.05, **P < 0.01, ***P < 0.0001 significance levels. TSS, Transcription Start Sites. Source data are available for this figure.

**Figure S9.**
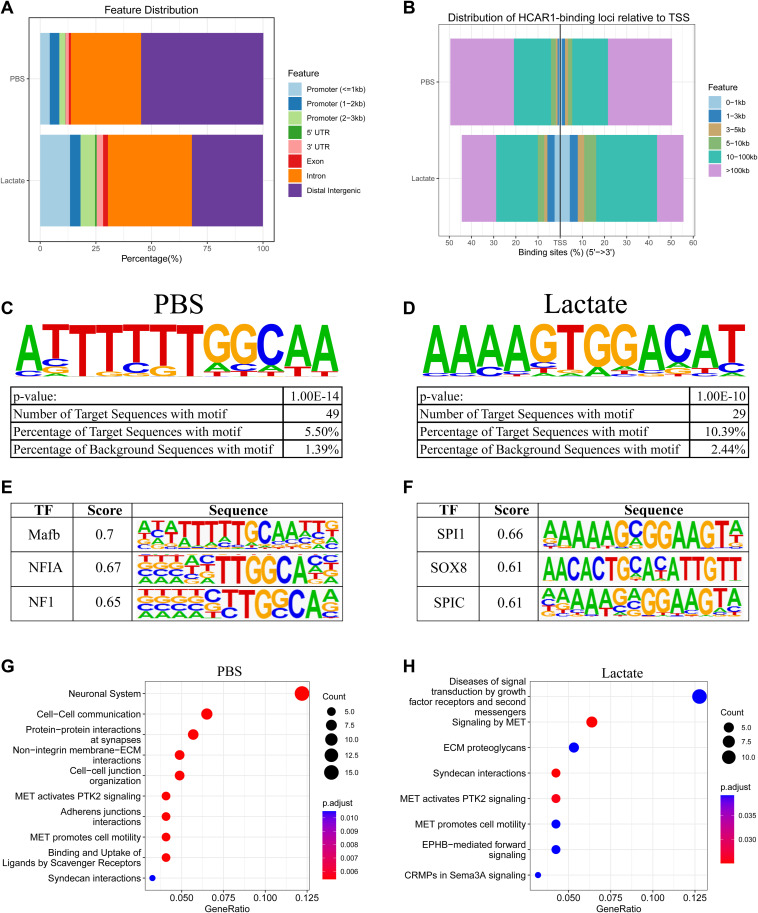
Extra analysis of ChIP-seq data. **(A)** Detailed feature distribution of HCAR1 occupancy on the genome. **(B)** Distribution of HCAR1 around TSS. **(C, D)** The most enriched binding motifs for HCAR1 in PBS and lactate-treated conditions. **(C, D, E, F)** The top three match to known motifs for transcription factors with the binding motifs of HCAR1 in PBS and lactate conditions (C, D). **(G, H)** The reactome pathway analysis for HCAR1-bound genes with PBS and lactate treatment.

Since several chromatin remodeling factors were also detected in the interactome of N-HCAR1 (Fig 3B), the potential for direct gene/chromatin regulation (rather than signaling for downstream gene regulation) by HCAR1 prompted us to perform ChIP-sequencing of HCAR1 to identify genes that interact with the receptor. N-HCAR1 interacted with chromatin and approximately 260 genes were found to bind to HCAR1 upon stimulation with lactate, whereas the number of genes associated with the unstimulated receptor was higher (∼600) (Fig 5A; Supplemental Data 2). Less than 8% of the genes were shared between vehicle and lactate treatment (Fig 5A), inferring that a conformational change in the N-HCAR1 caused a genomic redistribution. We verified the interaction of a selected gene panel (such as *SERPINE1*, *HCAR*, *PTGER4*) that are interacting with N-HCAR1 with or without lactate treatment or the shared genes with ChIP-qPCR along with extra controls (Source Data 4A–C). Unstimulated N-HCAR1 mostly localized to gene deserts, whereas upon lactate stimulation it occupied gene segments with considerable increase in promoter occupancy (Figs 5B and S9A). A similar trend can be observed at a smaller scale within individual genes, where unstimulated N-HCAR1 distributes in an unorganized pattern around transcription start sites, contrasting with a precise reorientation at transcription start sites upon lactate stimulation (Figs 5C and S9B). Consistently, the putative binding motifs enriched in the unstimulated and in the ligand-activated conditions are completely different (Fig S9C–F). Furthermore computational analysis revealed that the promoters of HCAR1-bound genes are co-enriched with positive regulatory epigenetic markers upon lactate treatment including H3K9ac, H3K27ac and H3K4me3 (Dunham et al, 2012; Xu et al, 2014), but are devoid of compact chromatin marker H3K27me3 (Fig 5E). Accordingly, gene expression analysis for some of the genes highly enriched with HCAR1 based on our ChIP-seq analysis showed an HCAR1-dependent expression profile, abrogated by N-HCAR1 KD (Fig 5D). Altogether, data provide convincing evidence for direct gene regulatory function of N-HCAR1.

Supplemental Data 2. HCAR1 ChIP-sequencing dataset.

Ontological and gene set enrichment analysis revealed that whereas the enriched genes for unstimulated N-HCAR1 are mainly involved in general homeostatic processes, ligand-activated N-HCAR1 binds to genes that regulate various features of cellular migration (Fig 5F). However, a complementary analysis using the Reactome feature of both gene sets concentrate on pathways related to different migratory phenotype (Fig S9G and H). We performed cell migration assay to validate the role of the nuclear population of HCAR1 in cell migration (Fig 5). Correspondingly, HeLa cells devoid of total or N-HCAR1 exhibited defective migration (Fig 5G); similar observations were made on U251MG and A549 cells (Fig S8C and D). Thus N-HCAR1, distinct from its canonical signaling capacity, is able to directly interact and regulate gene expression, particularly those involved in cell movement.

### HCAR1 at the nucleus regulates a larger gene network than its plasma membrane counterpart

**Figure 6.**
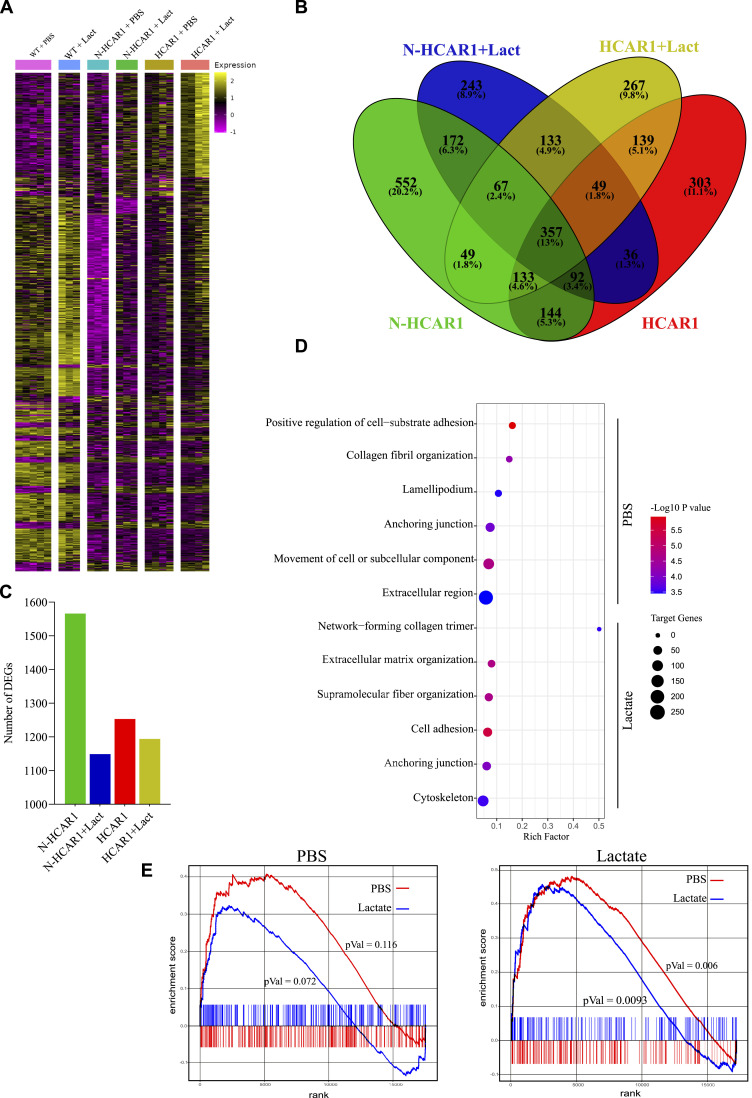
N-HCAR1 regulates a larger gene network than its plasma membrane/cytoplasmic counterpart. **(A, B, C)** Transcriptome of PBS and lactate treated (10 mM for 6 h) samples from scrambled shRNA, shHCAR1b and shHCAR1b+RNAi resistant δS305A HCAR1 cells. For validation of RNA-seq by qRT-PCR see Fig 5D. **(A)** Heatmap of significantly Differentially Expressed Genes (DEGs). **(B)** Venn diagram representing all DEGs in each line compared with shScrambled cell lines with their corresponding treatment. **(C)** Bar graph representing total number of all DEGs in each line compared with shScrambled with their corresponding treatment. **(D)** Ontological analysis of genes that were uniquely down-regulated only in HCAR1 nuclear KD cells with PBS or lactate treatments. **(E)** Waterfall plots representing overall general positive regulatory function of N-HCAR1 on gene transcription in N-HCAR1-bound genes (linking ChIP-seq and RNA-seq data). The expression values are extracted from RNA-seq data of HCAR1 nuclear KD cells with PBS and lactate treatments. The expression values represent WT condition to indicate expression level of genes regulated through N-HCAR1. The gene list is extracted from PBS-treated (left panel) and lactate-treated (right panel) HCAR1 ChIP-seq data. shScrambled PBS (n = 5), shScrambled lactate (n = 3), shHCAR1b PBS (n = 4), shHCAR1b Lacate (n = 4), shHCAR1b+ RNAi δS305A HCAR1 PBS and lactate (n = 3). Source data are available for this figure.

Our observations suggested that N-HCAR1 regulates gene expression through location-biased signaling and interactions with nuclear proteins and genes. We elucidated the transcriptomic network regulated by N-HCAR1 by performing RNA-seq (Fig 6A; Supplemental Data 3; Source Data 4D). Approximately 35% of all differentially regulated genes by HCAR1 were governed solely by N-HCAR1 and ∼26% through plasma membrane/cytoplasmic HCAR1 (Fig 6B and C). Although two-thirds of HCAR1 reside extra-nuclear, this higher level of gene regulation by N-HCAR1 (Fig 1F), highlights the importance of nuclear localization in this process. Stimulated and unstimulated conditions disclosed different transcriptomic profiles (Fig 6A and B); only ∼34% of genes were shared for stimulated and unstimulated conditions (Fig 6B). Interestingly, unstimulated N-HCAR1 regulates a larger gene network than other counterparts (Fig 6C), consistent with the ChIP-seq data.

Supplemental Data 3. HCAR1 trasncriptomic signature dataset.

In an attempt to determine if the N-HCAR1-gene complex, based on ChIP-seq results, culminates in gene expression or suppression, we aligned RNA-seq on the ChIP-seq data. Analysis revealed that most of the genes bound to N-HCAR1 (lactate stimulated or not [based on CHIP-seq]) were up-regulated by the N-HCAR1 (based on RNA-seq) (Fig 6E). Overall, findings suggest an unconventional function for N-HCAR1 in directly regulating gene expression through interactions involving protein/chromatin complexes, which are notably independent of lactate stimulation.

Ontological analysis of lactate-stimulated N-HCAR1-dependent transcriptome related to the migration pathways including anchoring junctions, network-forming collagen trimer and extracellular matrix organization (Fig 6D), consistent with ChIP-seq data (Fig 5F). Transcriptomic signature of unstimulated N-HCAR1 revealed other aspects of migration such as cell-substrate adhesion, collagen fibril organization and lamellipodium (Fig 6D). Gene Set Enrichment Analysis (GSEA) relative to migration ascertained N-HCAR1-dependent induction of genes involved in migration (Source Data 4E). Hence, stimulated and unstimulated N-HCAR1 coordinately promote expression of genes involved in different features of cell movement resulting in migration.

### N-HCAR1 promotes cancer growth and metastasis in vivo

**Figure S10.**
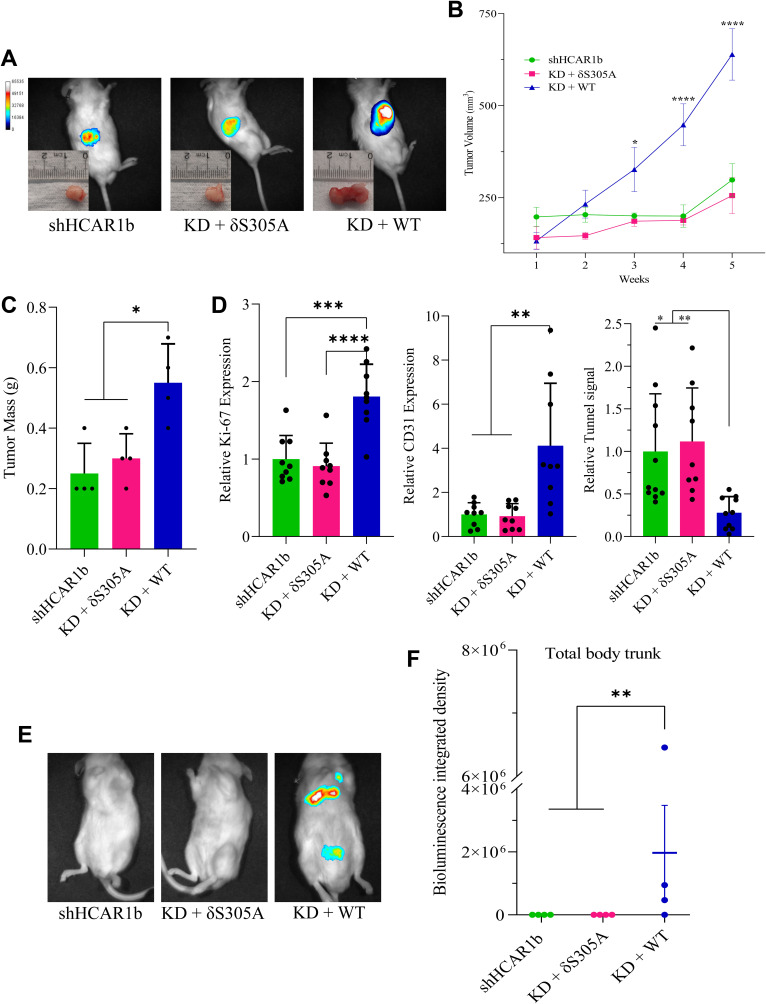
N-HCAR1 promotes cancer malignancy in vivo. **(A, B, C, D)** Subcutaneous injection of luciferase-expressing shHCAR1b cells, rescued or not with RNAi-resistant constructs δS305A or WT HCAR1, in NSG mice. **(A)** Representative images of in vivo luciferase signal and corresponding dissected tumors 5 wk after injection. **(B)** Tumor volume measurement. **(C)** Weight of dissected tumors 5 wk after injection. **(D)** Immunohistochemistry staining analysis from dissected tumors indicating expression levels (i.e., intensity) of Ki-67 and CD31 and cell death (TUNEL assay) relative to control samples. **(E, F)** Tail vein injection of the same cell lines as above in NSG mice. **(E)** Representative luciferase in vivo images indicating metastasis of the cells. **(F)** Bioluminescence intensity from body trunk of mice indicating metastasis. Each dot represents one mouse. Data in panel (B, C, F) are mean ± SEM and in panel are mean ± SD, from n = 4 biological replicates. The ANOVA was followed by Bonferroni post hoc correction test with *P < 0.05, **P < 0.01, ***P < 0.0001 significance levels.

HCAR1 has been shown to enhance cancer progression and metastasis in vivo (Roland et al, 2014; Stäubert et al, 2015; Lee et al, 2016), and N-HCAR1 mediates proliferation, survival, and migration of cancer cells in vitro (as shown in Figs 2E and F and 5G). We validated the role of N-HCAR1 in vivo by injecting luciferase-expressing HeLa cells subcutaneously in NOD/SCID/IL2Rγ null (NSG) mice. Proliferation of tumors was monitored by bioluminescent live imaging (Fig S10A). Tumor volume and mass markedly increased in mice injected with HCAR1-expressing WT-rescue cells compared with tumors silenced for HCAR1 and N-HCAR1 KD cells (δS305A rescue) (Fig S10B and C). Coherently, resected tumors expressing HCAR1 at the nucleus exhibited higher proliferation index (Ki-67) and endothelial density (CD31 positivity) consistent with angiogenesis, and less apoptosis (TUNEL staining), compared with tumors devoid of N-HCAR1 (Fig S10D; Source Data 5). To assess metastatic spread, HeLa cells were injected in the tail vein and metastatic tumor spread was monitored by bioluminescence. As seen with tumor volume, metastatic spread was observed only in HeLa cells expressing nuclear-intact HCAR1 (Fig S10E and F); no metastatic spread was detected in HCAR1 KD and N-HCAR1 KD cells. These data support the notion that the effects of nucleus-specific localized HCAR1 on different functions translate into promoting cancer growth and propagation in vivo.

## Discussion

GPCRs are involved in essentially every pathophysiological process; this has largely been thought to be based on their plasma membrane location and the receptor modality. Mounting evidence points to intracellular location of GPCRs and their downstream effectors, such that location-biased signaling is arising as a major concept in the field. Subcellular GPCRs and especially the nuclear ones are readily detected inside these organelles, other than on the lipid membrane of these organelles (Mohammad Nezhady et al, 2020). However, there is no report on the function of these GPCRs other than through their classical reliance on membrane-associated receptor function. Nonconventional activity of GPCRs would introduce the new concept of “location-biased activity” next to the location-biased signaling. Wheras the latter concept remains in its infancy, the former is a totally new one that has yet to be introduced, to the best of our knowledge. Accordingly, these understudied aspects of GPCR biology provide new avenues for therapeutic exploitation of this highly druggable receptor family; location bias (signaling and activity) expands on the physiologic effects of GPCRs which could explain enigmatic features of their involvement in a number of roles.

The requirement for intracellular lactate is observed for a number of HCAR1-dependent functions and the receptor’s mechanism of action is unexplained in these conditions (Wagner et al, 2015; Wagner et al, 2017; Raychaudhuri et al, 2019). Herein, we demonstrate several unprecedented molecular functions for a GPCR at the nucleus, which promote cancer malignancy, independent of conventional signaling activity. Whereas the nucleus contains one-third of the cellular reservoir of HCAR1, we provide unparalleled evidence that combined nuclear location-biased activity and signaling of a nuclear GPCR on gene regulation surpasses that exerted through its plasma membrane counterpart, underlining in this case the importance of nuclear HCAR1. Essentially, the nuclear HCAR1, other than its signaling activity, directly governs gene regulation via its interaction with the genome for various important functions including migration; the receptor also modulates critical processes such as protein translation and DNA damage repair through protein–protein interactions. All these processes were validated by functional assays at endogenously expressed level of HCAR1 (i.e., using scrambled shRNA and WT rescue); these effects were not observed in the mutant rescues excluded from the nucleus. Overall, these two points ascertain the validity of our high-throughput analysis and the specificity of N-HCAR1 involvement in these processes.

HCAR1 was found at the INM with analogous conformation to its plasma membrane counterpart and comparably capable of triggering classical G_αi_ and G_βγ_ protein-coupled signaling bursts of ERK and AKT activation in the nucleus. In addition, based on the interactome data for the N-HCAR1, it binds to transcriptional factors of different ATP-dependent chromatin remodeling complexes INO80, SWI/SNF, and ISWI (e.g., INO80b, SMARCC1, and BPTF, respectively) (Hasan & Ahuja, 2019); these interactions were observed with the stimulated and unstimulated receptor, suggesting a potential constitutive role of HCAR1 in modulating their activity. Whereas these chromatin remodelers are significantly misregulated in many cancers (Lu & Roberts, 2013; Prendergast et al, 2020; Li et al, 2021), N-HCAR1 activated by the higher concentration of lactate seen in tumors (Warburg effect) could alter their activity in favor of cancer promotion. Interestingly, N-HCAR1 also interacted with NSD1, a histone methyltransferase known to bind to different nuclear receptors (including estrogen, thyroid, retinoic acid, and retinoid receptors) (Tauchmann & Schwaller, 2021). Since NSD1 is frequently mis-regulated in cancers (Tauchmann & Schwaller, 2021), it is tempting to speculate that metabolic rewiring could cause epigenomic alterations in favor of cancer malignancy. Concordantly, our genome-wide association study suggests that N-HCAR1 could directly promote expression of genes involved in migration, potentially through such epigenetic modulations. We found that several genes such as *WNT3*, *SERPINE1*, and *CDH5* previously reported to be regulated through HCAR1 (Lee et al, 2016; Lee et al, 2018; Madaan et al, 2019; Khatib-Massalha et al, 2020), are indeed immunoprecipitated with N-HCAR1, suggestive of direct gene regulation. Direct gene regulation also seems to apply for *HCAR1* gene itself as well through lactate-stimulated N-HCAR1, consistent with the reported auto-induction of HCAR1 (Liu et al, 2009; Zhao et al, 2020; Ishihara et al, 2022). These wide-ranging properties of this GPCR are reminiscent of non-GPCR classical nuclear receptors, such as estrogen receptor (ERα) (Xu et al, 2021); ERα has been reported to possess RNA-binding capacity while regulating posttranscriptional expression and splicing of specific sets of genes. Although nuclear receptors are essentially transcriptional factors (compared with GPCRs), uncovering their non-transcriptional roles is a major discovery with potential therapeutic implications (Xu et al, 2021). Accordingly, emphasis on unconventional receptor functions of GPCRs could capitalize on development of inhibitors and allosteric modulators rather than solely focus on antagonists/agonists for therapeutic discovery.

The N-HCAR1 could detect its ligand, the lactate, in the luminal space of the nucleus and induce signaling cascades into the nucleus. Subtle changes in the concentration of the lactate in the nucleus, could rapidly induce a response via this location-biased signaling. LDH and lactate has readily been detected in the nucleus (Valvona et al, 2016; An et al, 2023). In addition, the produced ligand inside the cytoplasm could reach this luminal space and activate the intracytoplasmic signal transduction. Lactate transporters have been detected on the nuclear membranes (Merezhinskaya et al, 2004). This could serve as a mode for autocrine signaling without the need to secrete the ligand to the extracellular space, an intracrine pathway.

GPCRs for ligands, such as metabolites that are constantly present within the cell, are stochastically in either active or inactive states at any given time; the ratio of active to inactive state depends on the cellular concentration of the ligand (Felce et al, 2018). On the other hand, a single molecule GPCR stoichiometrically can simultaneously bind to G_α_, G_β_, GRKs, and arrestin (Gurevich & Gurevich, 2018). These interactions occur through the intracellular domains of a GPCR, and are distinct from their ability to form homo/heterodimers via their hydrophobic transmembrane domains (Wu et al, 2010), thus providing other docking sites for protein–protein interactions. These intricacies expose the abilities of GPCRs to form various protein complexes. Along these lines, one could envisage non-cylindrical conformations for a GPCR inside the nucleus with its hydrophobic domains deeply buried in protein complexes interacting with hydrophobic domains of other proteins (Mohammad Nezhady et al, 2020); such interactions could explain transcriptional and translational control of N-HCAR1 from within the nucleus.

The present study highlights the multifaceted functionality of GPCRs through its nuclear location and direct interaction with the genome. Nuclear HCAR1 provides an adaptive fitness for cells to respond to metabolic tweaks through intracellular ligands, as is the case for lactate which augments survival, proliferation and propagation of cancer cells, by acting via N-HCAR1. These myriad of roles for nuclear-resident HCAR1 might not be determinant for individual cellular processes it participates in, however, its collective functions on various processes convey a significant adaptation for cancer progression and malignancy, whereas providing an unprecedented dimension for GPCR biology.

## Materials and Methods

### Cell lines and treatments

HeLa (CCL-2 ATCC) and A549 (CCL-185 ATCC) were purchased from commercial vendors and maintained according to the manufacturers protocol in DMEM, 10% FBS and 1% Pen/Strep, U251MG cells were a kind gift from Dr. Hardy’s lab and maintained in EMEM + 2 mM Glutamine + 1% nonessential amino acids + 1 mM sodium pyruvate + 10% FBS and 1% Pen/Strep in a humidified incubator with 5% CO2 at 37°C. Stable cells were generated using appropriate drug selection (G-418; Puromycine) after plasmid transfection or viral transduction and were maintained in these antibiotic instead of Pen/Strep. A stock concentration of 500 mM lactate (in PBS and pH adjusted to 7.4) was used for cell stimulation and similar volume of PBS as vehicle was used as control. The data for end point phenotypic effects in HeLa cell are presented in the main figures and the data for end point phenotypic effects in A549 and U-251MG cells are presented in the supplementary figures.

Cell replication and survival were determined by enumerating live and dead cells using automatic countess cell counter (Thermo Fisher Scientific) using trypan blue exclusion assay (Thermo Fisher Scientific). Cells were treated with 20 μM 5FU or starved for 24 h for survival assay, and cell numbers were calculated before and after the treatments. Trypan blue was added in 1X ratio to the media containing cells, and each replicate was performed in quadruplicates to determine the number of live and dead cells. Each experiment was conducted at least in triplicates.

For immunofluorescence, cells were seeded on Poly-L-Lysine coated cover slips in a six well plates for overnight in incubator. For DNA damage, cells were irradiated with 1 Gy intensity using Faxitron CP-160 irradiator and let to recover for 4 h at 37°C in incubator, and then IF was performed on them.

### Plasmids and RNAi

Cells were transfected with human HCAR1 because of enhanced immunoreactivity to exogenous tag (such as Flag), enabling superior localization resolution and for immunoprecipitation, as well as for FAP and Bio-ID construct preparation. The cDNA encoding HCAR1 was PCR-amplified with encompassing appropriate restriction enzymes sites at both ends of the amplicon. The final product was gel-purified (GeneJET Gel Extraction Kit; Thermo Fisher Scientific), digested with the restriction enzymes and cloned into each vector. pCMV-Tag 2A (Agilent) was used for N-terminal flag tagging using EcoRI and HindIII flanking sites, pCDNA3.1-HCAR1-flag (Genscript) was used for C-terminal flag tagging. ICL3 and S305A mutations were generated using back-to-back primers on pCDNA3.1-HCAR1-Flag vector by Q5 Site-Directed Mutagenesis Kit (NEB). FAP fusion to HCAR1 was synthesized with insertion of HCAR1 using BsmI site into pMFAP-β1 vector (Spectragenetics), and BioID fusion was generated with HCAR1 insertion into flanking sites AccIII and AfIII in MCS-13X Linker-BioID2-HA (80899; Addgene) vector. All plasmids were sequenced to verify the correct insertion. Vectors were transfected into the cells using TranIT-X2 reagent (Mirus) according to the manufacturers protocol and grown on appropriate antibiotics to generate stable cell lines.

Lentiviral shRNA against HCAR1 targeting 3′UTR regions of the gene (shHCAR1a: GCTTTATTTCAGGCCGAATGA; shHCAR1b: GCTCTGACCTTCTTCAAATCT) and the scrambled shRNA were purchased from GeneCopoeia (Cat# LPP-HSH007585-LVRU6MP-100). Targeting the 3′UTR regions allowed us to use our previous plasmid constructs for rescue experiments.

### RNA isolation and quantitative PCR

RNA was isolated using either RiboZol (VWR) or RNeasy mini kit (QIAGEN) then was converted to cDNA using iScript (Bio-Rad) following manufacturer’s instructions. qRT-PCR was performed using SYBR green master mix (Bio-Rad) on Roche light cycler. HRP and 18S were used for normalization of the results (normalization to 18S is reported in the manuscript).

### Immunoblot and ELISA

Cells were lysed in RIPA buffer (Cell Signaling) and a cocktail of protease inhibitors (Roche) and protein concentration was measured with Bradford assay (Bio-Rad). Proteins were heated in reducing Laemmli sample buffer at 95°C and resolved in SDS–PAGE protein gel and transferred to PVDF membrane (Bio-Rad). Membranes were blocked using 5% BSA (Sigma-Aldrich) for 1 h and then incubated for overnight with the primary antibodies. Afterward membranes were washed 3X with TBST and incubated with HRP-conjugated secondary antibodies for 1 h and then were washed again and revealed by ECL (VWR) chemiluminescence.

ERK1/2 and AKT phosphorylation levels were measured with both western blot and ELISA kits (Abcam). Cells were treated overnight with PTX (300 ng/ml) or Gallein (20 μM) and then nuclei were isolated and suspended in 10 mM lactate or vehicle with rotation at 37°C for 15 min, washed with PBS 2X and then were lysed in either Laemmeli buffer for western blot or treated according to the manufacturers protocol for ELISA.

### Immunofluorescence (IF) staining

Wells of six-well plate with cells were rinsed three times with PBS, fixed in 4% formaldehyde (Sigma-Aldrich) for 10 min at RT, and then washed three times 5 min with PBS. Subsequently, cells were permeabilized with 0.1% Triton X-100 in PBS for 15 min at RT and blocked in 1% BSA in PBS/0.1% Tween-20 for 1 h at RT. Cover slips were incubated in a humid chamber with primary antibody for overnight at 4°C diluted in fresh blocking buffer. The antibody solution was washed with PBST (3x, 5 min) and samples were incubated with corresponding secondary antibody solution (Alexa-Fluor conjugated secondaries) for 2 h at RT in the dark. Cells were washed again 3 × 5 min in PBST, stained with 10 ng/ml DAPI in PBS, rinsed 2x in PBS, and then mounted in Prolong Gold mounting media (invitrogen) for subsequent microscopy acquisition.

### Wide-field microscopy

Cells were imaged on a Leica DMi8 microscope (Leica microsystems Gmbh, GER) using a HC PL APO 63x/1.40 oil objective and acquired using a Leica DFC9000 sCMOS camera (2048 × 2048 px, 16-bits, 216 MHz, no shading correction). Four-planes z-stacks with a step of 600 nm were obtained and projected on a single plane to prepare the figures. Control of the hardware was achieved using LASX software (Leica).

### Confocal microscopy

Slides were imaged with a Leica SP8 confocal microscope using a HC PL APO 63x/1.40 oil objective with pixel sizes of 30 × 30 μm; illumination was with laser lines: 488, 594, 647, and 680 nm. A combination of PMT and HyD hybrid detector (Leica) was positioned using LASX software such that there was no overlap between each channels based on control samples illumination. 200 nm stacked images were acquired for z-stacks using LASX (Leica microscopy licensed software) and analyzed using ImageJ (NIH). Imaris 9.9 software (Oxford Instruments) was used for 3D rendering.

### Transmission electron microscopy

Cells were fixed in 4% paraformaldehyde + 0.5% Glutaraldehyde in cacodylate buffer (0.1 M, pH7.2). After fixation, cells were washed 2 times in cacodylate buffer (5 min) and then in PBS. Permeabilization was performed with 0.2% Triton X-100 for 15 min, and then cells were blocked with PBST with 10% FBS for 1 h. Samples were incubated ON with primary antibody in blocking buffer at 4°C, and delivered to electron microscopy facility of faculty of medicine (Université de Montreal) for further processing and imaging. Primary antibody was detected with nanogold conjugated secondaries and silver enhancement. Thin sections with a thickness of 80 nm embedded in Epon were cut, and imaged with transmission electron microscope (CM120; Philips) equipped with a Gatan digital camera. Control samples were processes similarly. Quantification of nuclear to cytoplasmic ratio (Fig 1E) was performed using EM images by measuring the HCAR1 puncta from a single cell for 20 cells from two biological replicates and cells were chosen randomly.

### FAP pulse chase

*HCAR1* gene was cloned into pMFAP-β1 vector (Spectragenetics) and pulse-chase experiments were performed after generation of stable cell lines. Cell were treated with 100 nM βGREEN-np membrane impermeant fluorogen (Spectragenetics), which cannot enter the cell unless bound to FAP, and 1 min later cells were treated with 10 mM lactate (or PBS) for indicated time points. Afterward, cells were briefly washed with PBS and fixed with 4% paraformaldehyde, and the nuclei were stained with Hoechst 33342. βGREEN-np was excited and imaged using Alexa fluor 514 channel with Leica confocal microscopy. No nuclei with βGREEN-np were observed in the experiments. IF staining of stable cell lines without lactate treatment were performed as mentioned previously with Myc primary antibody against the Myc-tag in the N-terminus of HCAR1 and C-terminus of FAP.

### Nuclei isolation and staining

Isolating nuclei was performed as described previously (Nabbi & Riabowol, 2015). Briefly, cells were washed and resuspended in PBS plus protease inhibitor cocktail (Roche) and 1 mM PMSF. They were centrifuged (10,000 rpm - Hematocrit Rotor - for 10 s) and resuspended in PBS + protease inhibitor cocktail + 0.1% NP-40 and triturated for seven times with P1000 micropipette tip. Supernatant was collected (or removed) after centrifugation as cytoplasmic fraction. The pellet (containing nuclei) was subjected to second time trituration (five times) and centrifuged again to obtain pure nuclei fraction. After last centrifugation, supernatant was removed and nuclei were resuspended in PBS, Qiazol, or RIPA to for IF staining, RNA extraction, or Western blotting, respectively. The purity of nuclear fraction was assessed under the microscope and was validate by Western blotting.

Intact non-permeabilized nuclei were resuspended in PBS and mounted on Poly-L lysine coated cover slips for IF staining. For ONM permeabilization, nuclei were resuspended in 0.0008% Digitonin and rotated for 5 min at RT in microfuge tubes. Permeabilized nuclei were centrifuged, washed and resuspended in PBS + PI cocktail. For proteinase K digestion, nuclei were incubated at 37°C with rotation in 100 μg/ml proteinase K (Sigma-Aldrich) solution before or after ONM permeabilization with Digitonin. Nuclei were then washed 3x in 1% BSA + 5 mM PMSF solution and then further permeabilized with Digitonin for ONM permeabilization or 0.1% triton for INM permeabilization and then were subjected to IF staining.

### HCAR1 3D modeling

We analyzed the structure of HCAR1 in both active and inactive forms as described elsewhere (Kuei et al, 2011; Miszta et al, 2018), using the web service: https://gpcrm.biomodellab.eu/. Also we examined the inactive structure using recent AlphaFold (Jumper et al, 2021), and there was minimal differences between both models only in low confidence regions of the free c-terminus. Posttranslation phosphorylation site analysis was done using: https://www.phosphosite.org/, and described in (Hornbeck et al, 2015). Only phosphorylation residues with validated mass spectrometry data (HTP) were used for mutagenesis analysis.

### cAMP measurement in isolated nuclei

Isolated nuclei were resuspended in PBS with 10 mM lactate with rotation at 37°C for 10 min, nuclei were then counted using hemocytometer and were subjected to immunoassay cAMP Direct kit (Abcam) according to manufacturer’s protocol. Protein G-coated plated were used for the ELISA provided in the kit and measurements were performed with HRP development by measuring its OD at 450 nm with Clariostar plate reader.

### Bio-ID and mass spectrometry

*HCAR1* gene was cloned into MCS-13X Linker-BioID2-HA (80899; Addgene) vector. After validating the fusion protein has same localization pattern as the HCAR1 itself, stable cells were generated by antibiotic selection. Control samples were transfected stable cell lines with the empty vector. Cells were treated with Biotin (50 μM) and 10 mM lactate (or PBS) and incubated for ∼16 h in incubator. Nuclei were isolated and their purity was validated. Isolated nuclei were lysed with non-denaturing lysis buffer (20 mM Tris–HCl pH 8, 137 mM NaCl, 1% Nonidet P-40 (NP-40), 2 mM EDTA) plus PI cocktail (Boleslavska et al, 2022). Both experimental and control samples were analyzed in triplicates. The lysate was incubated with magnetic streptavidin MyOne Dynabeads (Thermo Fisher Scientific) at 4°C for ON with rotation. Beads were washed 5X in the lysis buffer and delivered to LC–MS/MS at IRIC Center for Advanced Proteomics Analyses, a Node of the Canadian Genomic Innovation Network that is supported by the Canadian Government through Genome Canada. Peptides were prepared with on-bead tryptic digestion based on previously established protocol (Turner et al, 2011; Elenbaas et al, 2023). Beads were washed 10 times with 50 mM Tris (pH 7.2), and afterward, were reconstituted in 50 mM ammonium bicarbonate with 10 mM TCEP (Tris [2-carboxyethyl] phosphine hydrochloride; Thermo Fisher Scientific), and vortexed for 1 h at 37°C. Chloroacetamide (Sigma-Aldrich) was added for alkylation to a final concentration of 55 mM. Samples were vortexed for another hour at 37°C. One microgram of trypsin was added, and digestion was performed for 8 h at 37°C. Samples were dried down and solubilized in 4% formic acid (FA). Peptides were loaded and separated on a home-made reversed-phase column (150-μm i.d. by 200 mm) with a 56-min gradient from 10 to 30% ACN-0.2% FA and a 600-nl/min flow rate on an Easy nLC-1000 connected to an Orbitrap Fusion (Thermo Fisher Scientific). Each full MS spectrum acquired at a resolution of 60,000 was followed by tandem-MS (MS–MS) spectra acquisition on the most abundant multiply charged precursor ions for a maximum of 3 s. Tandem-MS experiments were performed using collision-induced dissociation (CID) at a collision energy of 30%. The data were processed using PEAKS X (Bioinformatics Solutions, Waterloo, ON) and the Uniprot human database (20,349 entries). Mass tolerances on precursor and fragment ions were 10 ppm and 0.3 D, respectively. Fixed modification was carbamidomethyl (C). The data were visualized with Scaffold 4.0 (protein threshold, 99%, with at least 2 peptides identified and a false-discovery rate of 1% for peptides) (Inda et al, 2020; Khadivjam et al, 2023).

BioID data were analyzed first with Scaffold (Proteome Software Inc.) to produce quantitative values from normalized total spectra (Top 3 area based on Total Ion Count, TIC) for the amino acid sequences detected by mass spectrometry. Quantitative values for annotated proteins were then batch corrected using an empirical bayes framework (ComBat, SVA, https://rdocumentation.org/packages/sva/versions/3.20.0) and differential protein abundance between condition was assessed using MetaboAnalyst after quantile normalization, log transformation, and autoscaling (Pang et al, 2021). Statistical analysis using *t* test was performed to determine the proteins enriched in HCAR1 BioID cells compared with cells containing empty vectors (i.e., only the biotin ligase), in PBS and lactate treatment separately. Visualization of normalized data was done in R (version 4.1.0, 2021 The R Foundation for Statistical Computing) using gplots/heatmap.2 (https://CRAN.R-project.org/package=gplots), ggplot2 (https://CRAN.R-project.org/package=ggplot2), and EnhancedVolcano (Blighe et al [2022]. EnhancedVolcano: Publication-ready volcano plots with enhanced coloring and labeling. R package version 1.14.0). Pathway analysis of enriched proteins was performed on EnrichR (Xie et al, 2021) and Panther (Thomas et al, 2003). We additionally, used Significance Analysis of INTeractome (SAINT) (Choi et al, 2011) to identify proteins significantly labeled with HCAR1-BirA.

### Ribosomal profiling

Ribosome profiling was performed by sucrose gradient fractionation as described previously (Panda et al, 2017). Briefly, cells were treated with 10 μg/ml of cycloheximide (CHX) for 15 min at 37°C in the incubator to install ribosome disassembly. Cells were washed and resuspended in cold PBS containing CHX and PI cocktail, and then lysed in lysis buffer (20 mM Tris–HCl,100 mM KCl, 5 mM MgCl_2_, 0.5% Nonidet P-40) containing CHX, RNase inhibitor and PI cocktail. Lysate was cleared by centrifugation and equal amounts were layered on top of a cold sucrose gradient (10–60% gradient containing CHX, RNase inhibitor and PI cocktail). Gradients were centrifuged in Hitachi swinging ultracentrifuge (CP90NX) at 190,000*g* for 1.5 h at 4°C. Gradients were fractionated by piercing the bottom of sucrose gradient tube and the OD of collected fractions were measured at 254 nm spectrum. Ribosomal profile was plotted and area under the curve of each monosome subunit (40S, 60S, and 80S) and polysomes were measured for quantification of the ribosomal content.

### Protein translation rate measurement

Nascent protein synthesis rate was measured using Click-iT AHA Alexa Flour 488 protein synthesis HCS assay kit (Invitrogen) according to the manufacturer’s protocol. Equal number of cells was plated ON in a 96-well plate, and the media was washed out the next day and replaced with a methionine-free media containing L-azidohomoalanine (AHA) as the methionine analog, and incubated for 30 min. AHA is incorporated into proteins during protein synthesis in the methionine-free media. The amount of incorporated AHA is detected with a click chemical reaction by Alexa Flour 488. The intensity of Alexa Fluor 488 is adjusted with the intensity of DNA counterstain Hoechst 33342 and directly corresponds to the nascent protein synthesis rate.

### Co-IP

Cells were fractionated and cytoplasmic and nuclear fractions were lysed with non-denaturing lysis buffer plus PI cocktail. The lysates were then precleared with equilibrated protein G magnetic beads (Cell Signaling) for 1 h at RT with rotation. The precleared lysate was incubated with primary antibody O/N at 4°C. Prewashed magnetic beads were added to the immunocomplexes and incubated for 1 h at RT with rotation. Afterward, beads were isolated with magnetic separation rack and washed 5x with lysis buffer. Finally, beads were resuspended in 3x SDS sample buffer and incubated at 95°C for 5 min to elute the immunocomplexes. Elutes were analyzed by western blotting.

### ChIP-seq

Chromatin immunoprecipitation (ChIP) was performed using SimpleChIP Enzymatic Chromatin IP Kit (Cell Signaling) based on manufacturer’s protocol. Briefly, DNA-protein complexes were crosslinked using 1% final concentration of formaldehyde (Sigma-Aldrich) for 10 min at RT, and quenched with glycine (final concentration of 125 mM). Cells were then washed with cold PBS + PI cocktail, scraped into conical tubes and centrifuged to remove the supernatant. After isolation, nuclei were treated with micrococcal nuclease to digest the DNA, and then sonicated. Digested chromatin was analyzed by agarose gel. Chromatins were then incubated with immunoprecipitating antibody O/N at 4°C with rotation. Control samples were incubated with IgG antibody. ChIP-grade protein G magnetic beads were added to the IP reactions and incubated for 2 h at 4°C with rotation. Beads were then washed with low- to high-salt wash buffers and chromatin was eluted in elution buffer for 30 min at 65°C. Chromatins were reverse-crosslinked with NaCl and proteinase K and incubation at 65°C for 2 h. DNA was purified with spin columns, and analyzed by qPCR or sent to next generation sequencing. Samples were analyzed with bioanalyzer for quality control and single-end NGS was performed at IRIC genomic platform with Nextseq 500 illumina system. Samples were sequenced with a depth of ∼35 M per sample with 75 cycles. Both C- and N-terminus flag-tagged cells were used for ChIP-seq studies. Two samples from each terminus tagged cells were used for either lactate or vehicle (PBS); in total four samples per treatment group, and only shared genes in each group was used for further bioinformatic analysis.

ChipSeq fastq files were processed with default parameter using the ChipSeq pipeline from GenPipe (Bourgey et al, 2019). BAM files were visualized with IGV (Robinson et al, 2011) (A public access version is also available: PMC3346182). Peak files were analyzed using the ComputeMatrix function from deeptool (Ramírez et al, 2016) to determine distance relative to histone modification marks based on Broad Histone Helas Chip-seq data from the Encode project (Luo et al, 2020). ChIPseeker (Yu et al, 2015) was used to annotated the data and profile the binding peaks.

### Migration assay

Cells were cultured to reach a density of 90–100% confluency and then a wound was made by scratching the monolayer cells with sterile p200 pipette tips. Cells were washed and new media was added. Cells were imaged by phase contract microscopy right after scratch to measure the initial distance (t0), and later in indicated time points. Reduction of the scratched area due to the migration of the cells was measured as the rate of migration. Each experiment was conducted in triplicates and each time in multiple wells of the plates.

### Transcriptomic

Equal number of cells were seeded in 10 cm dish and let to grow a density of 70–80% confluency. Lactate was added to the final concentration of 10 mM (or equal volume of PBS) and incubated to 6 h. RNA was extracted with RNeasy mini Kit (QIAGEN). Samples were sent to IRIC genomic platform for analysis and sequencing. RNA integrity and quantity was validated with Bioanalyzer and then used for sequencing. Samples were sequenced with Nextseq 500 illumina system with a depth of ∼35 M per sample with single-end 75 cycles. Each sample was sequenced at least in triplicates.

RNAseq data were preprocessed using the RNAseq next-flow pipeline (Ewels et al, 2020) with the star_salmon aligner and the salmon pseudo-aligner (reference genome GRCh38). Gene counts were normalized and scaled to perform differential gene expression analysis between groups using Seurat after regressing for batch effect (Hao et al, 2021). Differentially expressed genes were further analyzed using fastGSEA (Korotkevich et al, 2021 *Preprint*) and enrichR (Xie et al, 2021).

### Sequencing

500 ng of total DNA for ChIP-sequencing or RNA was used for library preparation. DNA/RNA quality control was assessed with the Bioanalyzer Nano assay on the 2100 Bioanalyzer system (Agilent technologies) and all samples had a RIN above 9,5. For RNA, PolyA selection was done using Dyna Beads Oligo (dT) (Thermo Fisher Scientific). Library preparation was done with the KAPA DNA or RNA Hyperprep kit (Roche). Ligation was made with Illumina dual-index UMI (IDT). All libraries were diluted and normalized by qPCR using the KAPA library quantification kit (Cat no. KK4973; KAPA). Libraries were pooled to equimolar concentration. Sequencing was performed with the Illumina Nextseq500 using the Nextseq High Output 75 (1 × 75 bp) cycles kit. Around 30 M single-end PF reads were generated per sample for ChIP-sequencing and around 35 M for RNA sequencing. Library preparation and sequencing was made at the Institute for Research in Immunology and Cancer’s Genomics Platform (IRIC).

Raw base calls were converted to FASTQ files using bcl2fastq version 2.20 and allowing 0 mismatches in the multiplexing barcode. Before that, base calls had been obtained from the Illumina NextSeq 500 sequencer that runs RTA 2.11.3.0.

### Animal experiments

All animal procedures were approved by Institutional Ethic Committee of CR Sainte-Justine Hospital. NOD/SCID/IL2Rγ null (NSG) mice were obtained from Humanized mouse platform of CR-CHU Saint-Justine. Mice were housed in the sterile animal facility under pathogen-free conditions. 5-wk-old animals were separated for acclimatization and were injected with cells at 6 wk of age. HeLa cells (shScrambled, shHCAR1b, and KD+δS305A) were transduced with Renilla-Luciferase viral vectors and GFP-positive cells were sorted. Passage three of sorted cells were counted and 1 million cells were injected into animals. Mice were anesthetized with 2.5% isoflurane and cells were injected subcutaneously in the right flank after shaving and sterilizing the area for tumor growth monitoring, or injected into the tail vein for metastatic analysis of the cells. Two male and two female mice were used for each cell line. Animals were imaged once every 3 d for 5 wk. Mice were anesthetized and injected with D-Luciferin (150 mg/kg) 10 min before imaging. In vivo whole-body imaging was performed using Epi-Fluorescence and Trans-Fluorescence imaging system (OiS300; LabeoTech) and signal intensities were normalized and measured in radiance integrated density (photons ∣ s^−1^ ∣ sr^−1^ ∣ cm^−2^) using Fiji Macros. Animals were euthanized after 5 wk or at the study cut-off points (extreme abscess or 30% weight loss and morbid condition) and tumor and organs were harvested for histological analysis.

### Immunohistochemistry

Tissue samples were fixed in 10% Formalin O/N at RT, and then embedded in paraffine. Paraffin embedded blocks were cut to 5 μm sections and deparaffinized in Xylene and decreasing concentrations of ethanol. Antigen retrieval was done with Sodium Citrate Buffer (10 mM Sodium Citrate, 0.05% Tween, pH6) for 10 min with pressure cooker. Slides were washed with TBS/Triton X-100, and then blocked with 10% normal serum, 1% BSA in TBS for 2 h at RT. Incubation with primary antibody was done ON in 4°C, and endogenous peroxidase activity was blocked with 0.3% H_2_O_2_. Secondary HRP-conjugated antibody was used for detection. Slides were developed with DAB reagent and counterstained with DAPI. Samples were dehydrated, mounted, and visualized with Leica DMi8 wide-field microscope with monochrome color camera.

### Statistics

The number of samples per group, number of replicates and details of error bars are provided in the figure legends. Statistical tests were performed using GraphPad Prism 9.0 (GraphPad Software). For comparisons between two experimental groups, unpaired two-tailed *t* tests were used, and for comparison of three and more groups analysis of variance (ANOVA) was used followed by Bonferroni post hoc correction test with *P < 0.05, **P < 0.01, ***P < 0.0001 significance levels. Data are shown as the mean ± SD, except data in panel Fig S10B, C, and F which are mean ± SEM. Every dataset is composed of at least n ≥ 3 independent experiments. List of genes from high-throughput experiments were compared with Venny (Oliveros, 2007).

## Supplementary Material

Reviewer comments

## Data Availability

The Proteomic data from this publication have been deposited to the ProteomeXchange database and assigned the identifier PXD035882. The Transcriptomic and genomic data from this publication have been deposited to the GEO database and assigned the identifier GSE210470. Further methods and other data would be available upon request from the corresponding authors.
