# Supplementary material for: Unconventional receptor functions and location-biased signaling of the lactate GPCR in the nucleus
Source: Life Sci Alliance. 2025 Feb 4;8(4):e202503226. doi: 10.26508/lsa.202503226 (PMC11794946; doi:10.26508/lsa.202503226)
Supplement: Supplementary file 1 [file LSA-2025-03226_SdataF1.1.pdf]

a

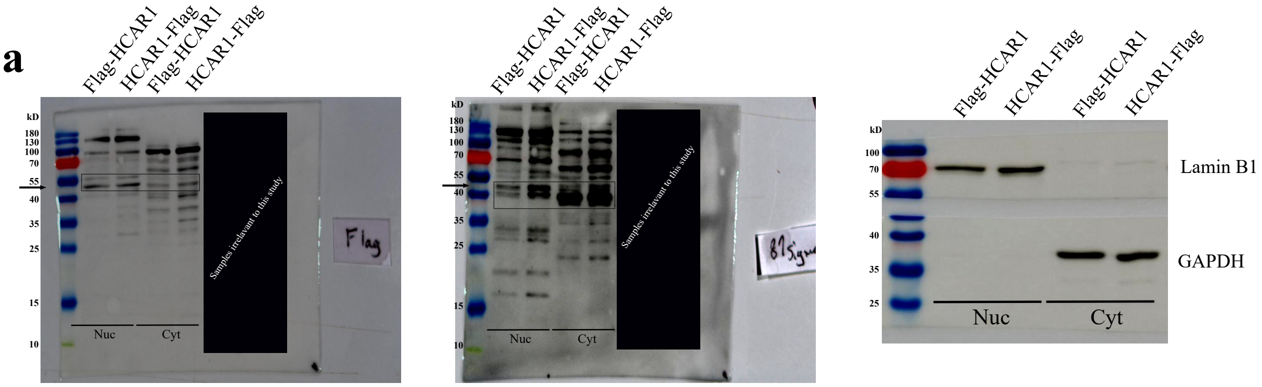

b

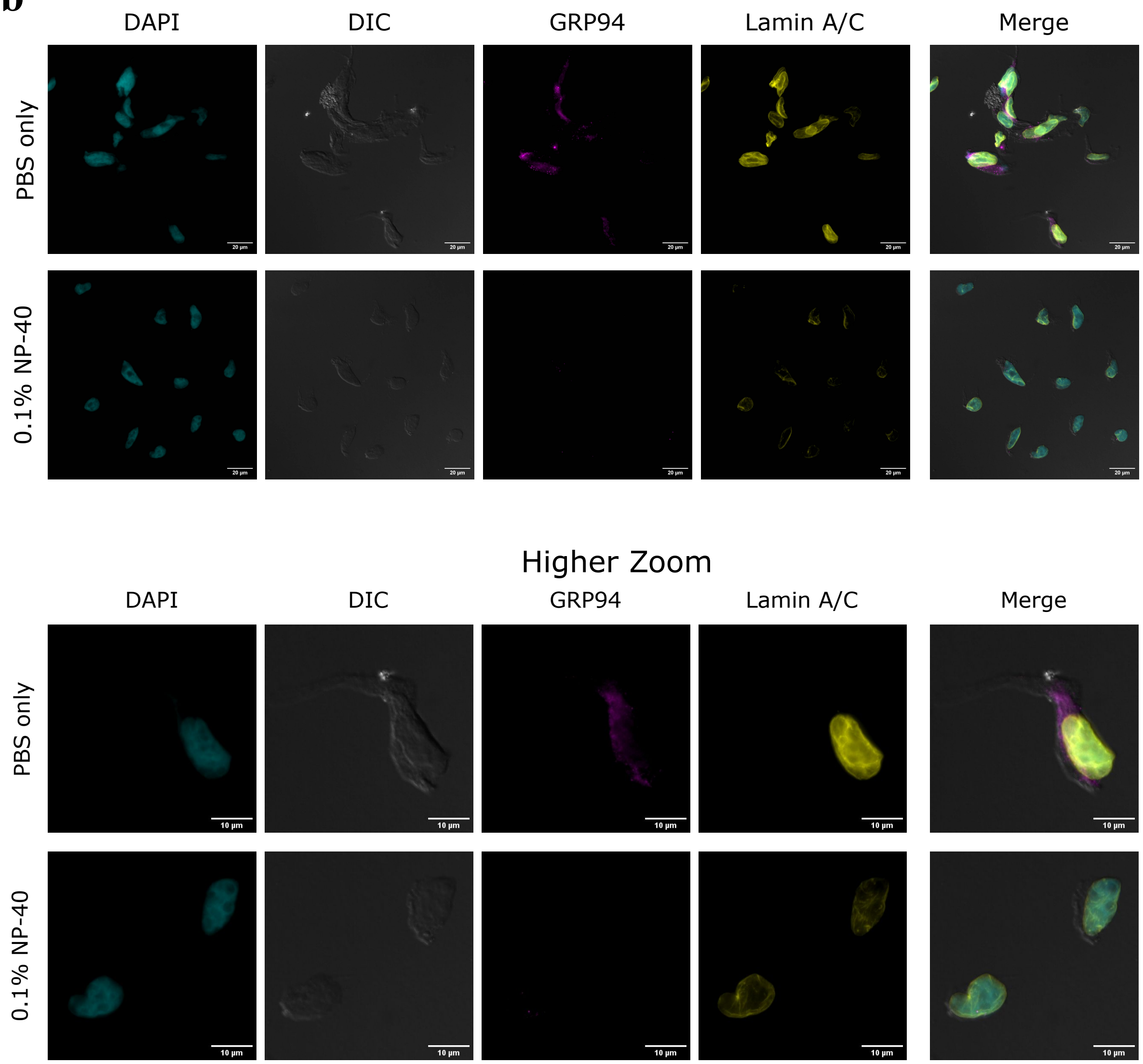

c

Modification Sites in Parent Protein, Orthologs, and Isoforms

Show Multiple Sequence Alignment

LTP	HTP		human		mouse
0	1	T219	QARMKKATRFIMVVA	T219-p	QARMRRAtRFIMVVA
0	1	I227	RFIMVVAIVFITCYL	S227-p	RFIMVVAsVFITCyL
0	1	Y233	AIVFITCYLPSVSAR	Y233-p	AsVFITCyLPSVLAR
0	1	S305	KPKQPGHSKTQRPEE	T305-p	KPKRPGRtKTRRSEE
