# Supplementary material for: Unconventional receptor functions and location-biased signaling of the lactate GPCR in the nucleus
Source: Life Sci Alliance. 2025 Feb 4;8(4):e202503226. doi: 10.26508/lsa.202503226 (PMC11794946; doi:10.26508/lsa.202503226)
Supplement: Supplementary file 2 [file LSA-2025-03226_SdataF1.2_F2.2_F3.pdf]

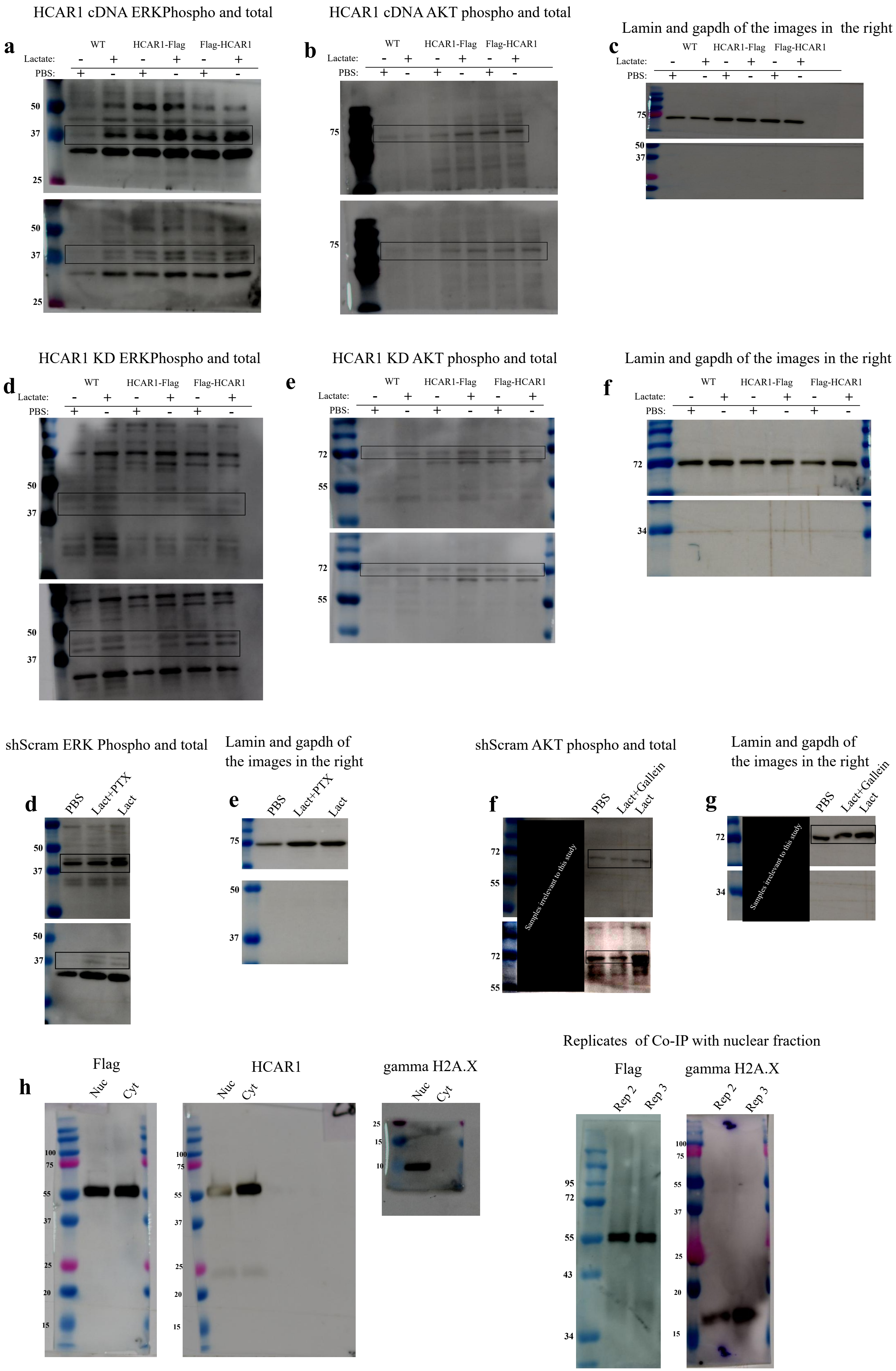
HCAR1 cDNA ERKPhospho and total
a
WT HCAR1-Flag Flag-HCAR1
Lactate: - + - + - +
PBS: + - + - + -
50
37
25
50
37
25
HCAR1 cDNA AKT phospho and total
b
WT HCAR1-Flag Flag-HCAR1
Lactate: - + - + - +
PBS: + - + - + -
75
75
Lamin and gapdh of the images in the right
c
WT HCAR1-Flag Flag-HCAR1
Lactate: - + - + - +
PBS: + - + - + -
75
50
37
HCAR1 KD ERKPhospho and total
d
WT HCAR1-Flag Flag-HCAR1
Lactate: - + - + - +
PBS: + - + - + -
50
37
50
37
HCAR1 KD AKT phospho and total
e
WT HCAR1-Flag Flag-HCAR1
Lactate: - + - + - +
PBS: + - + - + -
72
55
72
55
Lamin and gapdh of the images in the right
f
WT HCAR1-Flag Flag-HCAR1
Lactate: - + - + - +
PBS: + - + - + -
72
34
shScram ERK Phospho and total
d
PBS Lact+PTX Lact
50
37
50
37
Lamin and gapdh of the images in the right
e
PBS Lact+PTX Lact
75
50
37
shScram AKT phospho and total
f
PBS Lact+Gallein Lact
72
55
72
55
Samples irrelevant to this study
Lamin and gapdh of the images in the right
g
PBS Lact+Gallein Lact
72
34
Samples irrelevant to this study
h
Flag
Nuc Cyt
100
75
55
37
25
20
15
HCAR1
Nuc Cyt
100
75
55
37
25
20
15
gamma H2A.X
Nuc Cyt
25
15
10
Replicates of Co-IP with nuclear fraction
Flag
Rep 2 Rep 3
95
72
55
43
34
gamma H2A.X
Rep 2 Rep 3
100
75
55
37
25
20
15
