# Supplementary figures and images for: Unconventional receptor functions and location-biased signaling of the lactate GPCR in the nucleus
Source: Life Sci Alliance. 2025 Feb 4;8(4):e202503226. doi: 10.26508/lsa.202503226 (PMC11794946; doi:10.26508/lsa.202503226)

**a**

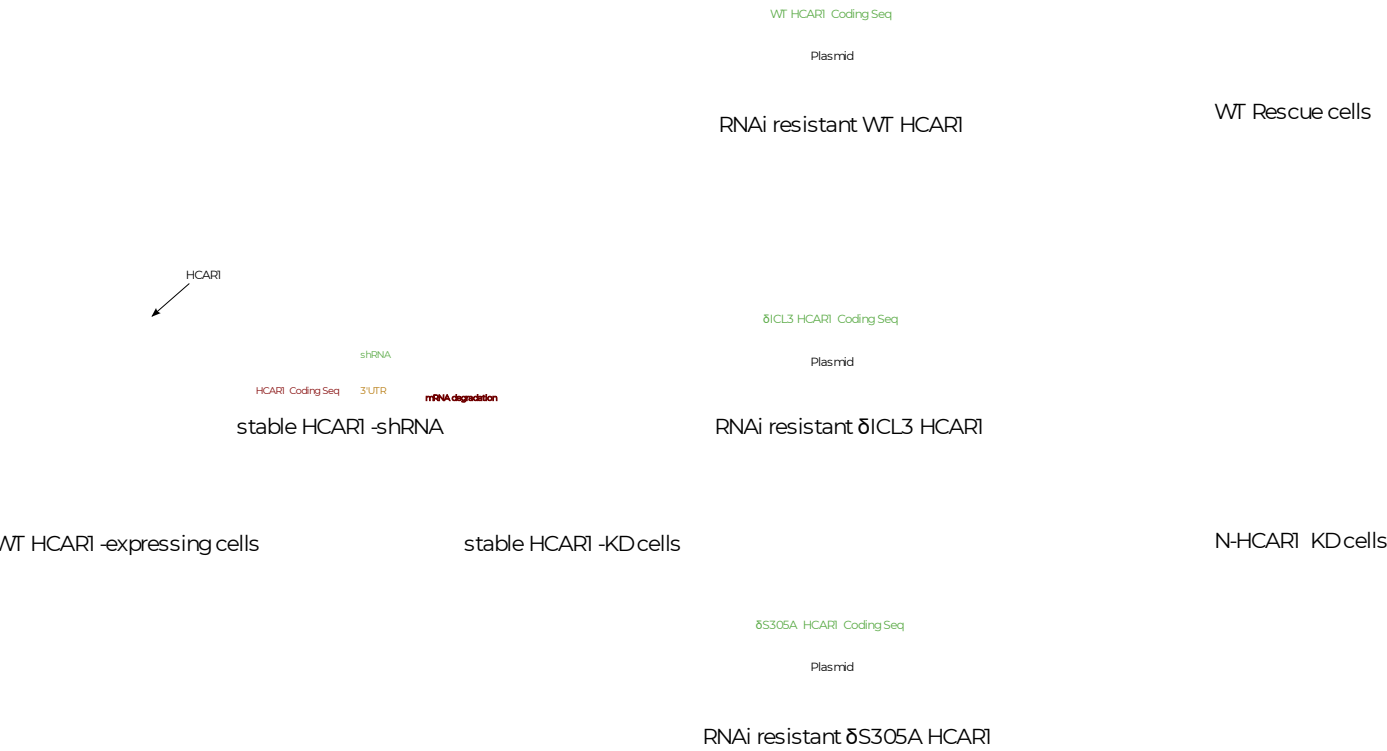

**b**

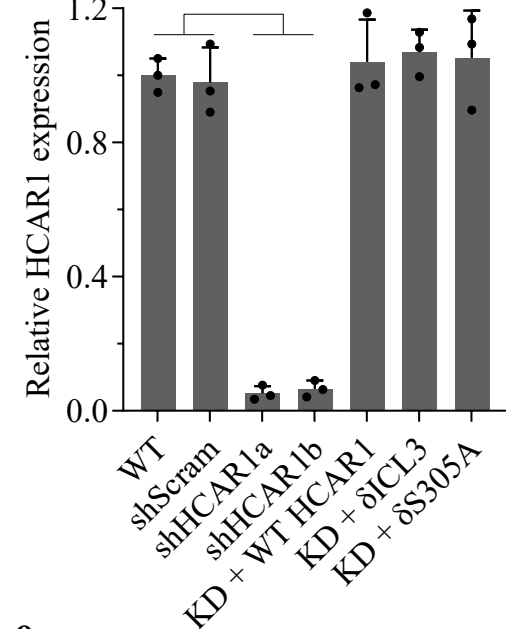

**c**

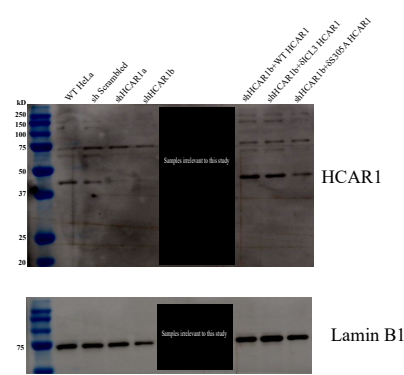

**d**

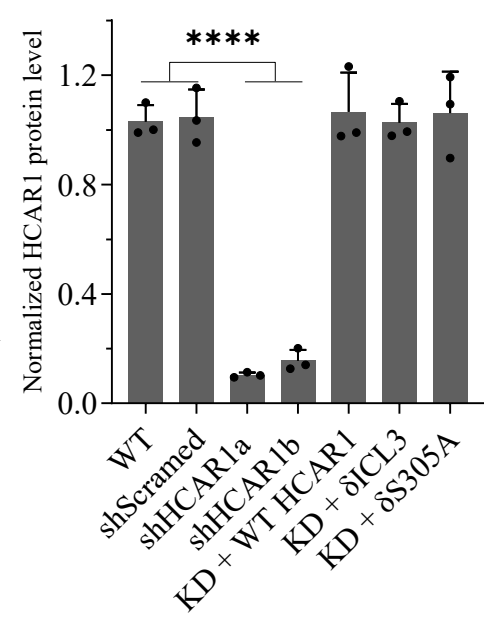

**e**

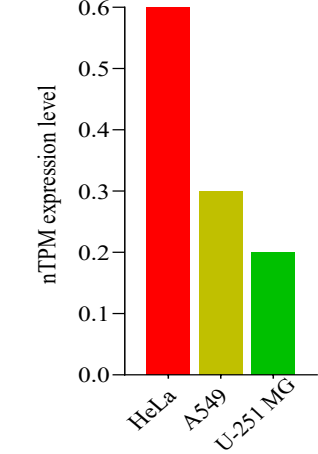

Supplement: Supplementary file 3 [file LSA-2025-03226_SdataF2.1.pdf]

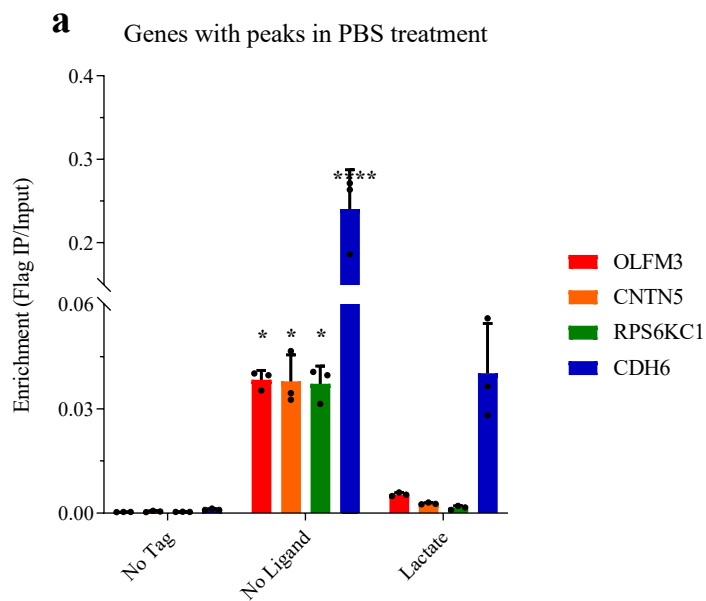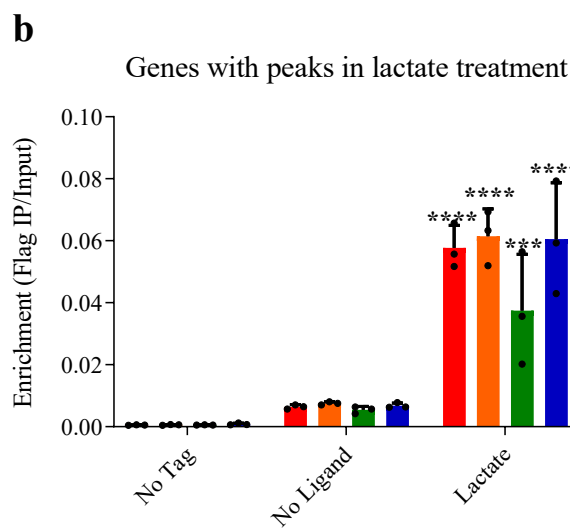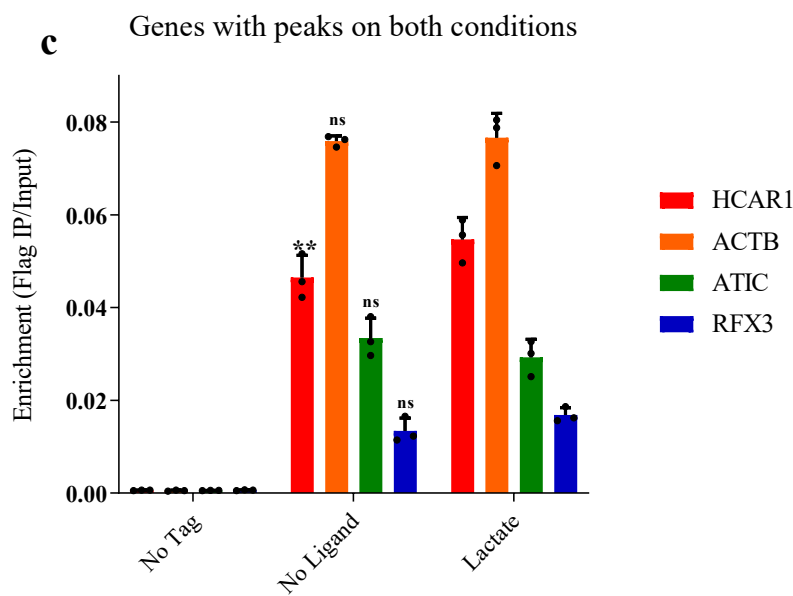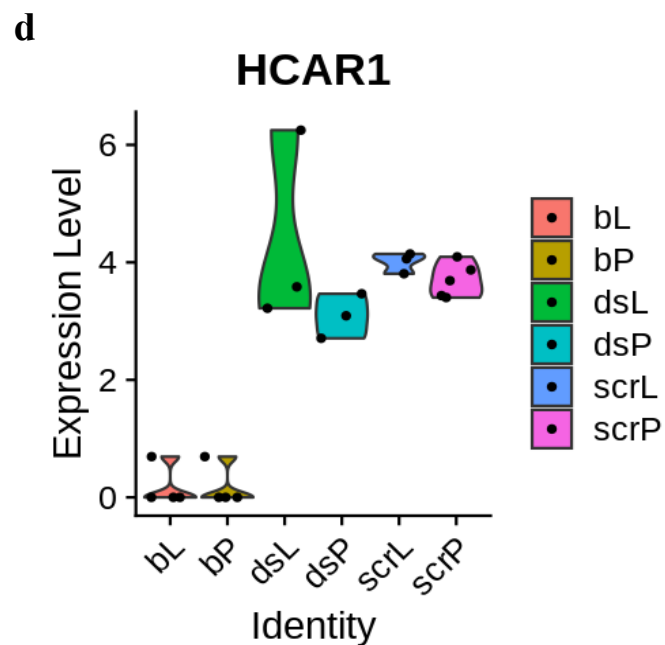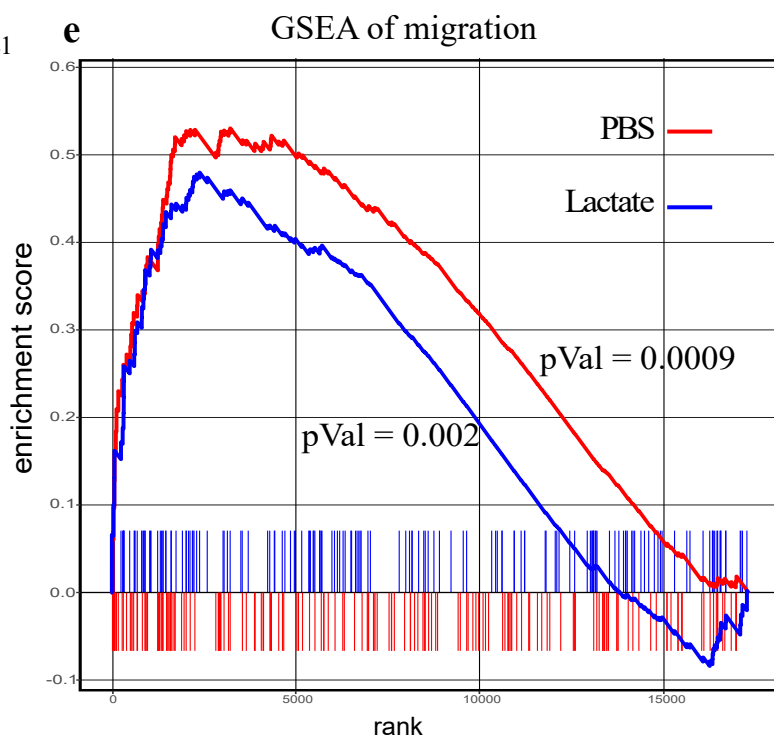

Supplement: Supplementary file 7 [file LSA-2025-03226_SdataF5.pdf]

shHCAR1b

KD +  $\delta$ S305A

KD + WT

a

Ki-67

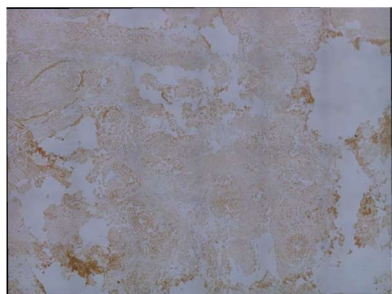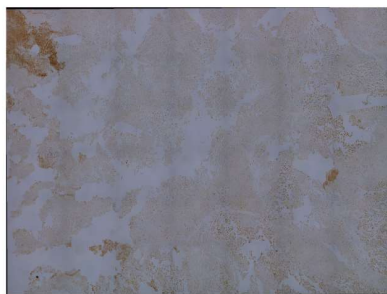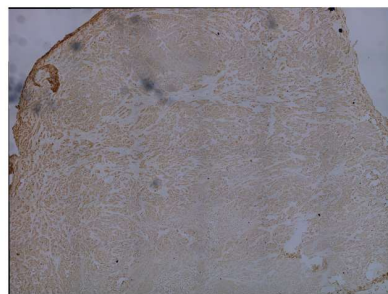

b

CD31

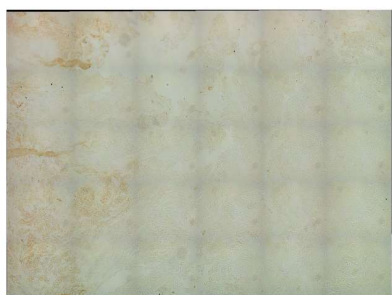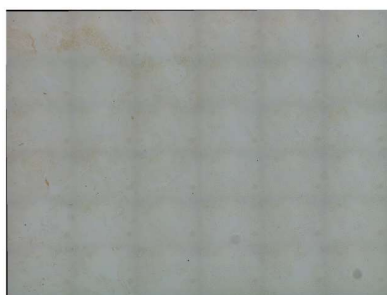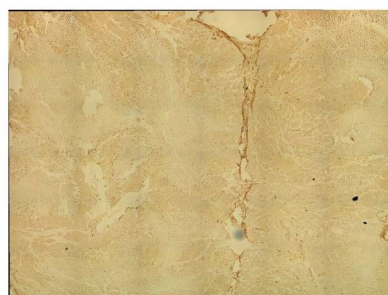

c

TUNEL

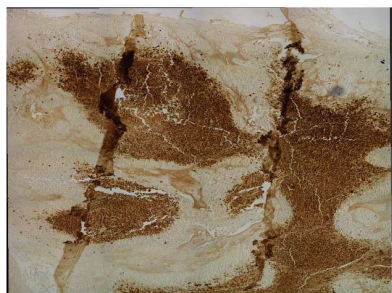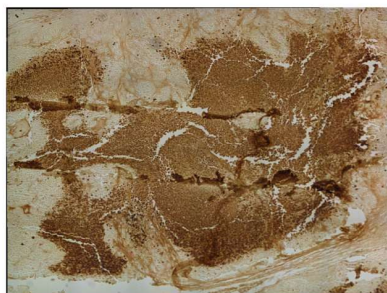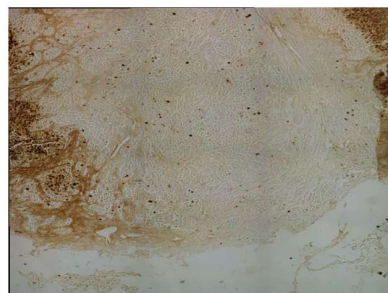

Supplement: Supplementary file 9 [file LSA-2025-03226_SdataF6.pdf]
