# Supplementary material for: Unconventional receptor functions and location-biased signaling of the lactate GPCR in the nucleus
Source: Life Sci Alliance. 2025 Feb 4;8(4):e202503226. doi: 10.26508/lsa.202503226 (PMC11794946; doi:10.26508/lsa.202503226)
Supplement: Supplementary file 5 [file LSA-2025-03226_TableS1.docx]

| **PBS** | PPM1G, NSD1, ASF1B, ASF1A, QCR2, ZFR2, SYDC, SYQ, ODB2, TECR, TR150, SYRC, REXO4, UBN2, MCA3, SAS10, ADT2, AIMP1, MIC19, P66B, AIMP2, SYK, SYEP, PLRG1, SYIC, THOC5, BPTF, SMCA5, SMRC1, THOC3, HSP7C, SYMC, NONO, LAS1L, PPIG, AT2A2, ZN512, PHF10, RRP12, THOC6, RPA43, RPA34, PTGES |
| --- | --- |
| **Lactate** | TITIN, RPP38, CLN6, SSRG, RRS1, H2AX, NU214, NOG2, S39A7, H2BFS, QCR8, MGST3, SYNEM, HS71L, VRK2, LTV1, CCD86, RPA12, EDC4, VDAC2, CR021, DDX3X, LAP2B, NOP58, BBX, VAPA, PGAM5, MMTA2,  RRP15 |
| **Shared** | ADT3, PHB2ATD3A |

Supplementary Table 1: List of proteins interacting with N-HCAR1 from BioID experiment.
